# Aggressive behaviour in childhood and adolescence: the role of smoking during pregnancy, evidence from four twin cohorts in the EU-ACTION consortium

**DOI:** 10.1017/S0033291718001344

**Published:** 2018-06-11

**Authors:** Margherita Malanchini, Emily Smith-Woolley, Ziada Ayorech, Kaili Rimfeld, Eva Krapohl, Eero Vuoksimaa, Tellervo Korhonen, Meike Bartels, Toos C.E.M. van Beijsterveldt, Richard J. Rose, Sebastian Lundström, Henrik Anckarsäter, Jaakko Kaprio, Paul Lichtenstein, Dorret I. Boomsma, Robert Plomin

**Affiliations:** 1 Social Genetic and Developmental Psychiatry Centre, King's College London, United Kingdom; 2 Department of Psychology, University of Texas at Austin, United States; 3 Institute for Molecular Medicine FIMM, University of Helsinki, Finland; 4 Department of Biological Psychology, Netherlands Twin Register, Vrije Universiteit, Amsterdam, The Netherlands; 5 Department of Psychological and Brain Sciences, Indiana University, United States; 6 Centre for Ethics, Law and Mental Health and the Gillberg Neuropsychiatry Centre, Gothenburg University, Sweden; 7 Department of Public Health, University of Helsinki, Helsinki, Finland; 8 Department of Medical Epidemiology and Biostatistics, Karolinska Institutet, Sweden

**Keywords:** Adolescence, aggression, cross-cultural, maternal smoking during pregnancy, meta-analysis, parental aggression, paternal smoking, perinatal

## Abstract

**Background:**

Maternal smoking during pregnancy (MSDP) has been linked to offspring's externalizing problems. It has been argued that socio-demographic factors (e.g. maternal age and education), co-occurring environmental risk factors, or pleiotropic genetic effects may account for the association between MSDP and later outcomes. This study provides a comprehensive investigation of the association between MSDP and a single harmonized component of externalizing: aggressive behaviour, measured throughout childhood and adolescence.

**Methods:**

Data came from four prospective twin cohorts – Twins Early Development Study, Netherlands Twin Register, Childhood and Adolescent Twin Study of Sweden, and FinnTwin12 study – who collaborate in the EU-ACTION consortium. Data from 30 708 unrelated individuals were analysed. Based on item level data, a harmonized measure of aggression was created at ages 9–10; 12; 14–15 and 16–18.

**Results:**

MSDP predicted aggression in childhood and adolescence. A meta-analysis across the four samples found the independent effect of MSDP to be 0.4% (*r* = 0.066), this remained consistent when analyses were performed separately by sex. All other perinatal factors combined explained 1.1% of the variance in aggression across all ages and samples (*r* = 0.112). Paternal smoking and aggressive parenting strategies did not account for the MSDP-aggression association, consistent with the hypothesis of a small direct link between MSDP and aggression.

**Conclusions:**

Perinatal factors, including MSDP, account for a small portion of the variance in aggression in childhood and adolescence. Later experiences may play a greater role in shaping adolescents’ aggressive behaviour.

## Introduction

Maternal smoking during pregnancy (MSDP) is a major public health concern. Although the incidence of MSDP has been decreasing in the last two decades, in 2010, 12.5% of Danish, 16.5% of Norwegian, 6.9% of Swedish and 15% of Finnish women still reported smoking while pregnant (Ekblad *et al.*, 2014). Consequently, understanding the effects of MDSP on offspring's health and behaviour remains a priority for research and society (Smedberg *et al.*, 2014).

As well as being associated with adverse birth-related outcomes (e.g. low birth weight and pre-term delivery; Knopik *et al.*, 2016), MSDP has been associated with more externalizing problems in offspring, including conduct disorder (Gaysina *et al.*, 2013) and attention deficit hyperactivity disorder (ADHD) (He *et al.*, 2017), and psychiatric conditions with externalizing components such as schizophrenia and bipolar disorder (Quinn *et al.*, 2017; Sutin *et al.*, 2017). Although the association between MSDP and externalizing problems in offspring is well documented, questions about mechanisms underlying the observed relation remain, particularly concerning the existence of a direct link between MSDP and externalizing.

Alternative hypotheses suggest that socio-demographic factors (e.g. low maternal education and young maternal age) and socio-economic characteristics (e.g. low household income) that often co-occur with MSDP account for the observed link. Such hypotheses are consistent with epidemiological research showing an increased incidence of MSDP in younger mothers, mothers who are single, and mothers from low socio-economic backgrounds (Ekblad *et al.*, 2014; Smedberg *et al.*, 2014). Monshouwer *et al.* (2011) found that after accounting for socio-demographic and socio-economic characteristics, pregnancy-related factors and a parental history of externalizing problems, the association between MSDP and offspring’ externalizing behaviour in adolescence was not significant. However, other studies found that the association between MSDP and offspring externalizing behaviour remained significant beyond socio-demographic and pregnancy-related characteristics (Ekblad *et al.*, 2010; Cornelius *et al.*, 2011; Gaysina *et al.*, 2013). This evidence has been interpreted as consistent with the existence of a direct relation between MSDP and behavioural outcomes in offspring.

The link between MSDP and offspring's externalizing behaviour may reflect an additional confound: A shared genetic propensity for smoking and externalizing problems. Adolescents and adults who smoke also show increased levels of externalizing problems (Korhonen *et al.*, 2008; Matuszka *et al.*, 2017; Conway *et al.*, 2017). If parents who are predisposed towards smoking are also more likely to share a predisposition towards externalizing problems, the link between MSDP and offspring's externalizing may be a product of these inherited co-morbid predispositions or of an interplay between genetic predisposition and environment.

Different strategies have been adopted to examine the genetic co-morbidity between smoking and externalizing. One strategy involves investigating the effects of paternal smoking. Consistent with the observation of pleiotropic effects between smoking and aggression (Dolan *et al.*, 2016), in the absence of a causal effect between MSDP and aggression, associations of equal effect size would be expected between maternal and paternal smoking and offspring's externalizing problems. In fact, both parents are equally as likely to pass on genes that predispose to both smoking and externalizing, so that a greater effect of maternal than of paternal smoking would point to the existence of a specific effect of MSDP. Exploring the role of paternal smoking partly addresses an additional confound: the impact of second-hand smoking. Mothers who smoke during pregnancy are more likely to have a partner who smokes (Knopik *et al.*, 2012), introducing the possibility that exposure to paternal second-hand smoking during gestation may be as harmful as MSDP.

Although exposure to second-hand smoking during pregnancy has been linked to increased developmental delay in offspring (Lee *et al.*, 2011), paternal smoking did not account for the effect of MSDP on offspring hyperactivity (Keyes *et al.*, 2014) and other externalizing outcomes (Dolan *et al.*, 2016). However, not all evidence is consistent, as some research has failed to find a significant difference between the effects of maternal and paternal smoking on children's externalizing behaviour (Langley *et al.*, 2012).

Studies have explored the role of a shared genetic predisposition directly by accounting for the role of parental externalizing problems. One study found that the effects of MSDP on offspring's ADHD were fully accounted for by maternal ADHD (Agrawal *et al.*, 2010), while another study reported that maternal and paternal history of antisocial behaviour did not account for the association between MSDP and offspring's antisocial behaviour (Estabrook *et al*., 2016). Consistently, maternal psychiatric history was observed to not fully account for the link between MSDP and bipolar disorder in offspring (Talati *et al.*, 2013).

A further line of research has leveraged differences within families in exposure to MSDP (Kuja-Halkola *et al.*, 2014; D'Onofrio *et al.*, 2016). Skoglund *et al.* (2014) found that the association between MSDP and offspring ADHD was progressively attenuated as confounding factors shared within extended families (differentially exposed cousins) and nuclear families (differentially exposed siblings) were taken into account. A consistent pattern of attenuated effects was observed for the association between MSDP and severe mental illness in offspring (Quinn *et al.*, 2017). When comparing siblings discordant for exposure to MSDP the association between MSDP and offspring's substance use was not significant (D'Onofrio *et al.*, 2012).

Other studies have observed a direct link between MSDP and offspring's externalizing problems after accounting for within-family confounds. Marceau *et al.* (2018) found that the effect of MSDP on variation in offspring's ADHD remained significant even after accounting for within-family factors and suggested that the effects of MSDP may be best captured when hyperactivity and impulsivity are assessed across the population rather than when considering severe diagnosis or symptoms count. Two further investigations supported a direct link between MSDP and offspring antisocial behaviour (Paradis *et al.*, 2017) and conduct disorder (Estabrook *et al.*, 2016) after accounting for within-family confounds.

Altogether, the evidence is inconclusive with respect to whether the link between MSDP and externalizing behaviour persists after accounting for potential confounds. Moreover, the effect size of the association remains a debated issue. A possibility that should be considered is that inconsistencies in study designs and the measures adopted, variation in sample sizes, as well as potential cross-cultural differences, may be related to discrepancies in the literature.

The current study overcomes limitations that have characterized previous work by providing a comprehensive investigation into the association between MSDP and one, more targeted and normatively assessed, component of externalizing: aggression. Combining data from four cross-cultural longitudinal samples, we created a harmonized measure of aggression; overcoming the limitation of inconsistencies in measurement that may have resulted in mixed findings. Our first aim was to obtain a meta-analytic estimate of the effect size of the association between MSDP and offspring aggression. While several studies have investigated the association between MSDP and other aspects of externalizing problems, the specific link between MSDP and aggression has received little attention. The second aim of the current study was that of exploring the role of potentially confounding perinatal and socio-demographic measures in the MSDP-aggression association. This second meta-analysis was repeated for males and females separately to explore gender differences in the MSDP-aggression association, which remain an outstanding issue in the literature. Our third aim was that of accounting for the potential confounds of exposure to second-hand smoking and shared genetic comorbidity by controlling for the effect of paternal smoking on the MSDP-aggression association. Lastly, the fourth aim was that of directly addressing the potential role of a shared genetic liability by accounting for parental aggressive behaviour.

## Methods

### Participants

Participants come from four large developmental European twin samples, which collaborate in the EU-**A**ggression in **C**hildren: Unravelling Gene-Environment Interplay to Inform **T**reatment and **I**nterventi**ON** strategies (ACTION) project (http://www.action-euproject.eu/): the Twins Early Development Study (TEDS), The Netherlands Twins Register (NTR), the FinnTwin12 (FT12) study, and the Childhood and Adolescent Twin Study of Sweden (CATSS). Across all samples, one twin from each pair was randomly selected for the analyses to control for non-independence of observations. Data from 30 708 individual twins were analysed. For every age group, only the cases for which data was present for both perinatal factors (first collection wave) and aggressive behavior were included, final *N*s are reported in the sections below. Power calculations (online Supplementary Table S1) indicated that the samples were adequately powered (>80%) to detect the observed small effects.

The *TEDS* (Haworth *et al.*, 2013) sample is an ongoing developmental twin study that has followed more than 10 000 families of twins from birth. All families with live twin births in England and Wales between 1994 and 1996 were contacted by the Office of National Statistics on behalf of the study. The present study includes data collected from parents at first contact (when the twins were 1–2 years-old, *N* = 13 360), when the twins were 9 (*N* = 3415) and 12 (*N* = 2847), as well as twin-reports at age 16 (*N* = 2174).

The *NTR* (Bartels *et al.*, 2007; van Beijsterveldt *et al.*, 2013) is an ongoing developmental study at Vrije Universiteit, Amsterdam. Twins in the study were born between 1987 and 2004. Twins are recruited after birth and followed longitudinally. Parents report on their twins’ development, behaviour and cognition and on socio-demographic characteristics until the age of 12. From the age of 14 onwards, the twins complete self-report measures. The present study includes NTR data collected from parents soon after the twins were born (*N* = 14 944), at age 10 (*N* = 9995) and 12 (*N* = 8492), and from self-report at ages 14 (*N* = 4532) and 16 (*N* = 3098).

The *FT12* sample (Kaprio, 2006, 2013) is a prospective longitudinal study of five sequential cohorts of Finnish twins. The study started when the twins were 12 and is ongoing, with the twins now in their early 30s. All the twins born between 1983 and 1987 were invited to participate in the FT12 study. A subset of 40% of the twins was selected to participate in a more intensive study. The selected sample was assessed using parent reports and twin reports at age 12 (*N* = 2813), 14 (*N* = 1037) and 17 (*N* = 2114). Retrospective reports on perinatal measures (*N* = 5050) were collected from the parents when the twins were 12.

The *CATSS* sample was established in 2004, and it includes twins born in Sweden since 1992. The parents of 9-year-old twins were invited to complete interviews about their twins (with the exception of the first 3 years of the study that invited parents of 9–12 year-olds). Data on the first interview with parents were available for more than 27 000 individuals. Twins and parents were invited to take part in follow-up data collections when the twins were 15 and 18 years-old (see Anckarsäter *et al.*, 2011 for detailed information on the sample and data collected at every wave). The present study includes data collected from parental reports at age 9 (*N* = 13 500) and from self-reports at ages 15 (*N* = 4133) and 18 (*N* = 2974). At first contact parents also reported on perinatal measures (*N* = 13 458).

### Measures

#### Aggression in late childhood and adolescence

Different measures of aggression obtained from several informants (parents, teachers, and self-reports) were collected across samples and ages. Table 1 describes all the measures collected to index aggressive behaviour.

**Table 1.** Measures of aggression collected across the four samples

Sample	Age	Measures	Informant
Twins Early Development Study (TEDS)	9	Strengths and Difficulties Questionnaire (SDQ, Goodman, 1997);	Parent reports
		Reactive and Proactive Aggression Questionnaire (RPAQ, Dodge and Coie, 1987)	
	12	SDQ	Parent reports
	16	SDQ; Self-Report Delinquency Scale (Elliott *et al.*, 1985)	Self-reports
Netherlands Twins Register (NTR)	10	Child Behaviour Check List (CBCL; Achenbach and Edelbrock, 1991)	Parent report
	12	CBCL	Parent report
	14	Youth Self Report (YSR, Achenbach *et al.*, 1995; Wesseldijk *et al.*, 2017)	Self-reports
	16	YSR	Self-reports
FinnTwin12 (FT12)	12	Multidimensional Peer Nomination Inventory (MPNI, Pulkkinen *et al.*, 1999)	Parent reports
	14	MPNI	Self-reports
	17	MPNI	Self-reports
Childhood and Adolescent Twin Study of Sweden (CATSS)	9	Autism – Tics, ADHD and other Comorbidities (A-TAC; Hansson *et al.*, 2005)	Parent reports
	15	RPAQ; SDQ; and Self-Reported Delinquency questionnaire (SRD; Junger-Tas *et al.*, 1994)	Self-reports
	18	SRD; and Life History of Aggression scale (LHA, Coccaro *et al.*, 1997)	Self-reports

For this project, we created a harmonized measure of aggression for four age categories: (1) parent-reported aggression at age 9–10 (including data from TEDS, NTR and CATSS); (2) Parent-reported aggression age 12 (all samples); (3) Twin-reported aggression at age 14–15 (NTR, FT12, and CATSS); and (4) Twin reported aggression at age 16–18 (all samples). The harmonized measures were at first created based on similarity of items. The items included were those that described comparable behaviours across all the samples for which data were present for a specific age category. For example at age 9–10 data were available in three samples: TEDS, NTR, and CATSS. The CATSS sample included the item ‘*S/he often argues with adults*’, the TEDS sample the item ‘*Often argues with adults*’, and the NTR sample ‘*Argues a lot*’. These items were considered to tap the same behaviour, and included in the measure aggression at age 9–10. Similarly, at age 14–15 we had information available from three samples: CATSS, NTR, and FT12. The CATSS sample included the item: ‘*I fight a lot. I can make other people do what I want*’; the NTR sample the item: ‘I fight a lot’; and the FT12 sample the item: ‘*When angry, I might hit, push, kick, or throw something at the person* ’. These three items were judged as describing comparable behaviours. All items included in the harmonized measures loaded substantially onto one factor. The online Supplementary Table S2 includes detailed information on all items that were included in each measure and their factor structure, factor loadings, and internal validity.

#### Perinatal measures

Information on *MSDP* in all four cohorts is given in Table 2, together with the percentage of mothers who reported smoking during pregnancy, and those mothers who reported smoking heavily while pregnant. Additional details on the MSDP measure are provided in the online Supplementary Material.

**Table 2.** Measure of MSDP across samples, percentage of mothers who reported smoking, and percentage of mothers who reported smoking heavily (Level 3) during pregnancy

Sample	Levels of the MSDP measure	% of MSDP*	% of heavy MSDP*
TEDS	1 = no cigarettes	18.9% (*N* = 2595)	5.5% (*N* = 755)
	2 = 10 or < cigarettes per day		
	3 = 11+ cigarettes per day		
NTR	1 = no cigarettes	18.9% (*N* = 2942)	2.6% (*N* = 404)
	2 = <10 cigarettes per day (or pipe)		
	3 = 10+ cigarettes per day		
FT12	1 = no cigarettes	11.4% (*N* = 303)	3.6% (*N* = 95)
	2 = <10 cigarettes per day		
	3 = 10+ cigarettes per day		
CATSS	1 = no cigarettes	11.5% (*N* = 1558)	3.9% (*N* = 526)
	2 = <10 cigarettes per day		
	3 = 10+ cigarettes per day		

*Note:* MSDP = maternal smoking during pregnancy; * = out of the total *N*.

The smoking variables were also re-coded and dichotomized in order to explore the potentially non-linear effect of MSDP on offspring's aggressive behaviour. A first dichotomous variable was created, indicating whether mothers had smoked or not during pregnancy (irrespectively of the amount they smoked). A second dichotomous variable was created indexing whether mothers had smoked heavily during pregnancy (10 or more cigarettes per day).

Other perinatal measures available in every cohort included *maternal age when the twins were born, maternal and paternal highest educational qualification, and birth weight*. In addition to these measures, *TEDS* collected information on gestational age, length of breastfeeding (in days), drinking during pregnancy (0 = did not drink, 1 = yes 1–2 units per week, 3 = yes, more than 3 units per week), and whether mothers had experienced any major stress during pregnancy (present for a subsample of mothers). *NTR* also provided information on gestational age and length of breastfeeding (measured in days). *FT12* also had data on gestational age.

Additionally, *NTR* and *FT12* provided information on *paternal smoking*. In *NTR* information on paternal smoking during pregnancy was collected shortly after the twins were born together with reports of MSDP. The measure was originally coded on four levels (as described for MSDP), and was recoded into a three-level measure where (1) = ‘Did not smoke during the twin pregnancy’; (2) ‘Smoked less than 10 cigarettes per day/or smoked a pipe’; (3) ‘Smoked more than 10 cigarettes per day’. In *FT12*, information on paternal smoking was collected asking fathers whether they had ever smoked. The item asked: ‘*Do you smoke, or have you ever smoked, cigarettes regularly?*’ to which participants responded with 1 = ‘No’ or 2 = ‘Yes’. For the purpose of the present analysis responses were recoded into 0 = ‘No’ and 1 = ‘Yes’.

In addition to perinatal socio-demographic and family measures, *TEDS* included one item assessing aggressive parenting style. The 1-item measure was collected as part of a larger questionnaire on parenting style, and asked parents to rate on a scale from 1 to 3 (where 1 = ‘Rarely or Never’ to 3 = ‘Often’) the following statement: ‘*When my child misbehaves I give her/him a smack*’. We used this as a proxy for *parental aggression*.

### Analytic strategies

#### Linear and hierarchical regression analyses

Linear regression analyses were conducted in order to obtain the meta-analysed effect sizes: In the first meta-analysis, which explored the total main effect of the association between MSDP and aggression, *r* was obtained from correlation analyses. For the second meta-analysis (subsequently repeated for males and females separately), which explored the effect of MSDP on offspring's aggression after accounting for all other perinatal measures, the *r* coefficient was obtained from hierarchical regression analyses. All perinatal measures were entered at the first step in the hierarchical regression, while MSDP was introduced at the second step; the square root of the *R*^2^ change between Step 1 and 2 was used as the effect size. Hierarchical regressions also addressed our third and fourth aim.

#### Three-level meta-analyses

We conducted three-level meta-analyses using the ‘metafor’ package in R, applying Restricted Maximum Likelihood Estimation (REML). The 3-level meta-analyses were conducted in order to account for the complex data structure of the present investigation. In fact, as well as individuals (Level 1) being clustered within age group (Level 2), they were also clustered within four twin cohorts (Level 3; Pastor and Lazowski, 2017). In order to avoid the problematic standard error formulation of a correlation in its standard form (Alexander *et al.*, 1989), correlations were transformed using Fisher's *z*. Each effect size was then weighted by its inverse variance weight in order to allocate greater weight to larger samples, the standard error for the estimated common effect size is a function of such weights. The Fisher's *z*-transformed *r* coefficients were transformed back into their *r* form for the presentation of the results. In order to estimate statistic heterogeneity, we used the *I*^2^ statistics, a transformation of *H* (Higgins and Thompson, 2002) which describes the proportion of total variation that is due to heterogeneity. As well as calculating the cumulative *I*^2^, indicating the total variance due to true heterogeneity, within a three-level meta-analytic framework we were able to calculate the proportion of variance due to between cluster heterogeneity (each study, *N* = 13), and that due to within cluster heterogeneity (each twin cohort, *N* = 4). An example of the R code used to conduct the three-level meta-analyses and calculate the total and relative *I*^2^ is included in the online Supplementary Material.

## Results

### Descriptive statistics and correlations

Descriptive statistics for all measures of aggression are reported in online Supplementary Table S3 and for the perinatal measures in online Supplementary Table S4. The correlations between aggression and perinatal measures, including MSDP are reported in online Supplementary Table S5. Associations between perinatal measures and measures of aggression were weak and inconsistent across studies. One exception was MSDP, which yielded significant albeit modest correlations with aggression across ages and across studies (between *r* = 0.040 and *r* = 0.122).

### Hierarchical regressions

Hierarchical regressions explored the prediction from perinatal measures to later aggression in each of the four samples. All perinatal measures with the exception of MSDP were entered in the regression first. MSDP was entered at the second stage. This allowed to obtain *R*^2^ values for the prediction from MSDP to aggression independent of the effect of other perinatal measures.

With the exception of two assessments, MSDP remained a significant predictor of aggression throughout childhood and adolescence, although the effects were small, with *R*^2^ ranging from 0.001 to 0.008. The results of hierarchical regressions are reported in the online Supplementary Tables S6–S9.

Hierarchical regression analyses also investigated dose-specific effects, namely whether heavy MSDP was incrementally linked to offspring's aggression. Table 2 presents the percentage of mothers who reported smoking and smoking heavily while pregnant. Results (online Supplementary Table S10) showed that heavy MSDP (smoking 10 or more cigarettes a day) did not incrementally predict aggressive behaviour, for the majority of assessments. Only in four cases, heavy MSDP explained a significant, but negligible additional proportion of variance (*R*^2^ = 0.001–0.002).

### Meta-analyses

**Fig. 1.**
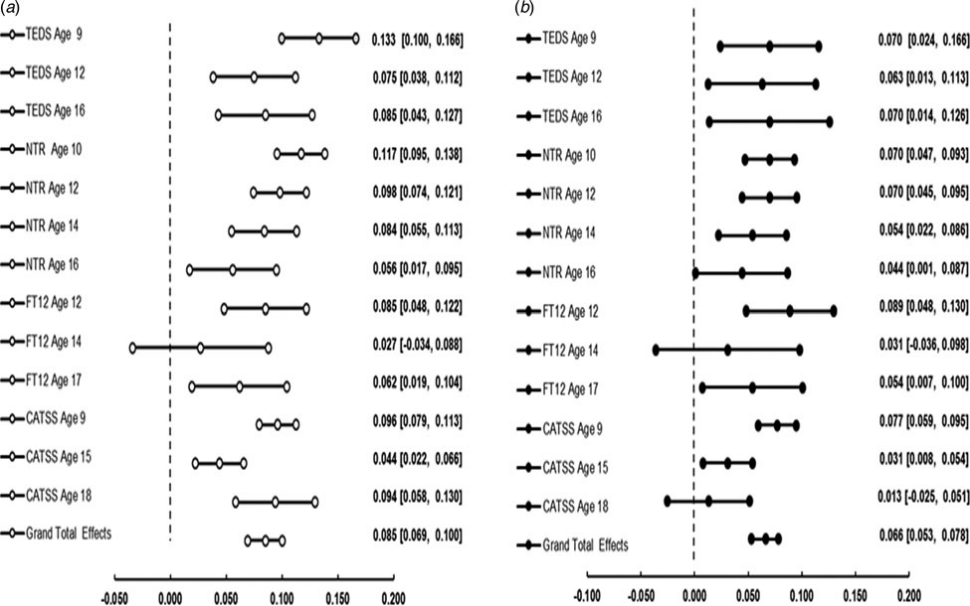
Forest plots showing the results of the meta-analyses exploring (a) the main effect of the correlation between MSDP and offspring's aggression in childhood and adolescence, and (b) the effect size of the association between MSDP and offspring's aggression after accounting for the confounding effect of other perinatal measures. The central dot in every line indicates the mean estimate, while the two dots at the extremities of each line indicate the lower bound and upper bound 95% confidence intervals (CIs).

To obtain a sample size-weighted estimate of the association between MSDP and aggression observed across all the samples at all ages, we conducted a three-level meta-analysis of the observed associations between MSDP and child and adolescent aggression. Results are presented in Fig. 1a. MSDP was significantly associated with child and adolescent aggression, *r* = 0.085 (95% CIs = 0.069–0.100; *z* = 10.90, *p* < 0.0001). The *I*^2^ statistics indicated that 69% of the variance was due to true heterogeneity, of this true heterogeneity 69% (*I*^2^ = 68.914) was due to between cluster heterogeneity and 0% (*I*^2^ = 0.000) to within cluster heterogeneity.

A second three-level meta-analysis explored the association between MSDP and aggression after accounting for all perinatal factors. The meta-analysed effect sizes were obtained by taking the square root of the change in *R*^2^ values, consequently obtaining *r*, before conducting the meta-analysis. Results (Fig. 1b) showed that, although the effect was reduced, MSDP remained a significant predictor of aggression in childhood and adolescence (*r* = 0.066, 95% CIs = 0.053–0.078, translating to 0.4% of the variance). The *I*^2^ statistics indicated that of the total 44% of the variance that was due to true heterogeneity, 44% (*I*^2^ = 43.951) was due to between cluster heterogeneity and 0% (*I*^2^ = 0.000) was due to within cluster heterogeneity.

This second meta-analysis was repeated for males and females separately. Results, reported in online Supplementary Table S11 showed that estimates were highly consistent across sexes, with the confidence intervals around the estimate for males (*r* = 0.073, 95% CIs = 0.061–0.086) overlapping with those observed for females (*r* = 0.067, 95% CIs = 0.055–0.079).

An additional meta-analysis estimated the combined effect of the prediction from all perinatal measures (except MSDP) to aggression (online Supplementary Table S12). Perinatal measures explained 1.1% of the variance in aggression across all cohorts and ages (*r* = 0.112, 95% CIs = 0.086–0.137). The *I*^2^ statistics indicated that of the total 76.76% of the variance that was due to true heterogeneity, 42.1% (*I*^2^ = 42.100) was due to between cluster heterogeneity and 34.7% (*I*^2^ = 34.669) was due to within cluster heterogeneity.

### Potential mechanisms explaining the association between MSDP and aggression

#### Paternal smoking

Results from the hierarchical regressions in the two samples that included a measure of paternal smoking are presented in online Supplementary Table S13 for NTR (paternal smoking during pregnancy) and in online Supplementary Table S14 for FT12 (ever smoking in fathers). Paternal smoking did not account for the association between MSDP and aggression.

#### Parental aggression

To test an additional mechanism that might underlie the association between MSDP and later aggression we conducted two further hierarchical regressions. These analyses controlled for aggressive parenting strategies (conceptualized here as a proxy for aggression in parents). The analyses were conducted in TEDS at ages 9 and 12. Results (online Supplementary Table S15) indicate that parental aggression did not account for the prediction from MSDP to offspring aggression.

## Discussion

The present study provides a comprehensive investigation of the association between MSDP and a harmonized measure of aggressive behaviour in childhood and adolescence. By exploring four research questions across four large epidemiological cohorts from Europe, we provide evidence for a small but significant link between MSDP and offspring aggression.

We observed a significant association between MSDP and aggression that was consistent across all cohorts at all ages. The association was small, but of interest, particularly when compared with the inconsistencies observed in the relations between aggression and the other perinatal measures considered in the present study.

Later experiences have been found to play a greater role in shaping aggressive behaviour in late childhood and adolescence. For example, negative parent-child communication during adolescence has been found to relate to an increased risk of aggressive behaviour with moderate effect sizes (Wallenius and Punamäki, 2008). Similar results were observed for parental hostility and family economic pressure experienced in early adolescence (Williams *et al.*, 2007). The findings of the present research also point us in this direction; in fact, we observed that a proxy measure for aggressive parenting strategies assessed in late childhood accounts alone for a similar amount of variance in aggression as all perinatal factors combined.

We examined if the association between MSDP and aggression could be due to other characteristics of mothers who smoke during pregnancy. We showed that the relation between MSDP and aggression in later childhood and adolescence is not a product of such confounding effects. In fact, although the association was slightly reduced, meta-analytical evidence showed that MSDP remained a significant predictor of aggression after accounting for several confounding perinatal measures, including maternal stress during pregnancy, gestation age, length of breastfeeding, and socio-demographic factors, such as parental education and occupation. This is in line with previous research findings (e.g. Gaysina *et al.*, 2013; Quinn *et al.*, 2017) and with the possibility of a small direct effect from MSDP to later externalizing problems. While a significant linear relation between MSDP and offspring aggression was found, we did not find that smoking heavily was associated with an increased risk. This is likely due to a lack of power as very few mothers reported smoking more than ten cigarettes per day while pregnant.

Since sex differences in the developmental trajectory of aggression have been consistently observed (Becht *et al.*, 2015), we investigated whether the association between MSDP and aggression differed between males and females. Our findings showing that the effects of MSDP on later aggression are comparable for males and females further support the hypothesis of a small direct link between MSDP and aggression.

Exposure to paternal smoking during pregnancy and later in development did not account for the link between MSDP and aggression. The majority of studies considering the effect of paternal smoking only focused on exposure during gestation (Roza *et al.*, 2009; Dolan *et al.*, 2016), and not on the potential effects of exposure to second-hand paternal smoking later in development. Our study, including a measure of paternal smoking not restricted to pregnancy, suggests that paternal smoking, during or after pregnancy does not account for the association between MSDP and aggression, providing additional support for the hypothesis of a small direct link.

Lastly, we considered the potentially confounding effects of parental aggressive behaviour in the MSDP-aggression association, showing that, while parental aggression was associated with offspring aggressive behaviour, this association did not account for the link between MSDP and later aggression. Our findings are in line with evidence showing that parental self-reports of antisocial behaviour did not account for the association between MSDP and antisocial behaviour in offspring (Estabrook *et al.*, 2016).

These findings point to the existence of a small direct link between MDSP and childhood and adolescent aggression that is remarkably consistent cross-culturally. Further evidence for and against our hypothesis can come from within-family studies, but to date these have found inconsistent results (D'Onofrio *et al.*, 2010; Kuja-Halkola *et al.*, 2014; Estabrook *et al.*, 2016; Marceau *et al.*, 2018; Paradis *et al.*, 2017; Quinn *et al.*, 2017).

Our results are consistent with the existence of a small direct link between MSDP and aggression and that this link may be reduced when family-wide factors are taken into account (e.g. Thapar *et al.*, 2009). These two propositions are not mutually exclusive. In fact, it is likely that both familial factors contributing to the intergenerational transmission of aggressive behavior, as well as a small direct link, contribute to the widely observed association between MSDP and externalizing symptoms, including aggression. A small direct link between MSDP and aggression is further supported by epigenetic evidence showing that MSDP is associated with alterations in DNA methylation and dysregulated expression of microRNA (Knopik *et al.*, 2012).

### Limitations

The current study presents a number of limitations. The first main limitation was the inability to capitalize on the genetic relatedness of the twin cohorts to inform our investigation. As perinatal measures, including MSDP, do not differ across twins, we were unable with the current data to investigate the aetiology of the observed associations. Other approaches, including DNA-based methods, such as genomic-relatedness-based restricted maximum likelihood (GREML; Yang *et al.*, 2011), which are not constrained by the within-family nature of the perinatal assessments may in future resolve this limitation. Relatedly, the second limitation was the inability to directly account for the familial confounds in the MSDP-aggression association, by for example examine differentially exposed relatives. The third limitation of the current study is the lack of in-depth phenotypic information on parental aggressive behaviour. Although the one-item measure included in the current investigation positively correlated with offspring aggression, this remains a very limited proxy for prenatal aggressive behaviour. Relatedly, we were not able to address the potential differences in a predisposition towards aggression between mothers and fathers who reported smoking during gestation. A fourth potential limitation is in the creation of the harmonized measure of aggression, which was based on the similarity between available items. Alternative harmonization techniques based on integrative data analyses may lead to more precisely defined harmonized phenotypes.

In conclusion, we found evidence for a small direct link between MSD and aggression across four large cohorts in the EU-ACTION consortium. The link was remarkably consistent across samples and sexes, and remained significant after accounting for possible confounding factors. Taken together our findings suggest that early environmental experiences have an only modest impact in shaping aggressive behaviour later in development, and point to the importance of exploring the role of later experiences, which is part of the ACTION future research plans.

## Supplementary material

For supplementary material accompanying this paper visit https://doi.org/10.1017/S0033291718001344. click here to view supplementary material
