# Supplementary material for: Aggressive behaviour in childhood and adolescence: the role of smoking during pregnancy, evidence from four twin cohorts in the EU-ACTION consortium
Source: Psychol Med. 2018 Jun 11;49(4):646–54. doi: 10.1017/S0033291718001344 (PMC6378412; doi:10.1017/S0033291718001344)
Supplement: Supplementary file 1 [file S0033291718001344sup001.doc]

**Aggressive behaviour in childhood and adolescence: the role of smoking during pregnancy, evidence from the Eu-ACTION study in four twin cohorts.**

**List of tables**

**Table S1**. Power calculation

**Table S2.** Items included in the harmonized measure of aggression at all ages and their factor loadings on the first principal component.

**Table S3.** Descriptive statistics for the measures of aggression across the four samples.

**Table S4.** Descriptive statistics for all perinatal measures across the four samples.

**Table S5.** Correlations between maternal smoking during pregnancy (MSDP), perinatal measures and offspring’s aggression in the Childhood and Adolescent Twin Study of Sweden (CATSS) sample (a), TEDS sample (b), NTR sample (c) and FT12 sample (d).

**Table S6.** Hierarchical regressions exploring the association between perinatal measures, MSDP and aggression in the Twins Early Development Study (TEDS) sample at age 9 (a), 12 (b) and 16 (c).

**Table S7.** Hierarchical regressions exploring the association between perinatal measures, MSDP and aggression in the Netherlands Twin Register (NTR) sample at age 10 (a), 12 (b), 14 (c) and 16 (d).

**Table S8.** Hierarchical regressions exploring the association between perinatal measures, MSDP and aggression in the FinnTwin12 (FT12) sample at age 12 (a), 14 (b), and 17 (c).

**Table S9.** Hierarchical regressions exploring the association between perinatal measures, MSDP and aggression in the Childhood and Adolescent Twin Study of Sweden (CATSS) sample at age 10 (a), 15 (b), and 18 (c).

**Table S10.** Hierarchical regressions exploring the effect of heavy MSDP in the TEDS (a), NTR (b), FT12 (c) and CATSS (d) samples.

**Table S11.** Meta-analysis exploring the effect of the association between MSDP and aggression after accounting for perinatal measures for males (a) and females (b) separately.

**Table S12.** Meta-analysis exploring the effect of the association between perinatal measures (excluding MSDP) and aggression.

**Table S13.** Hierarchical regressions exploring the effects of paternal smoking in the NTR sample

**Table S14.** Hierarchical regressions exploring the effects of paternal smoking in the FT12 sample

**Table S15.** Association between MSDP and aggression after accounting for aggressive parenting at ages 9(a) and 12 (b) in the TEDS sample.

**Detailed description of the measure of MSDP included on each twin cohort.**

**Example of R code used for the three-level meta-analysis.**

**Table S1.** Power calculation for all samples at all ages

| ***Sample*** | ***Predictors N*** | ***observed R^2^*** | ***p*** | ***sample*** | ***Power*** |
| --- | --- | --- | --- | --- | --- |
| TEDS 9 | 9 | 0.03 | 0.05 | 1814 | 0.9999 |
| TEDS 12 | 9 | 0.02 | 0.05 | 1515 | 0.9878 |
| TEDS 16 | 9 | 0.03 | 0.05 | 1206 | 0.9969 |
| NTR 10 | 6 | 0.03 | 0.05 | 7061 | 1.0000 |
| NTR 12 | 6 | 0.02 | 0.05 | 5865 | 1.0000 |
| NTR 14 | 6 | 0.01 | 0.05 | 3087 | 0.9949 |
| NTR 16 | 6 | 0.01 | 0.05 | 2073 | 0.9498 |
| FT12 12 | 5 | 0.02 | 0.05 | 2261 | 0.9999 |
| FT12 14 | 5 | 0.01 | 0.05 | 848 | 0.6028 |
| FT12 17 | 5 | 0.02 | 0.05 | 1764 | 0.9989 |
| CATSS 9 | 5 | 0.01 | 0.05 | 12078 | 1.0000 |
| CATSS 15 | 5 | 0.01 | 0.05 | 7181 | 0.9999 |
| CATSS 18 | 5 | 0.02 | 0.05 | 2634 | 0.9999 |

Note. *N* = maximum number of predictors included in the model.

**Table S2.** Items included in the harmonized measure of aggression at all ages and their factor loadings (FL) on the first principal component.

| **Age 9-10 Parent-reported aggression** | | | | | | | |
| --- | --- | --- | --- | --- | --- | --- | --- |
| CATSS | | TEDS | | NTR | | FT12 | |
| Item | FL | Item | FL | Item | FL | Item | FL |
| 1. Does s/he often argue with adults? | 0.70 | 1. Often argues with adults | 0.59 | 1. Argues a lot | 0.74 | Not available |  |
| 2. Does s/he often tease others by deliberately doing things that are perceived as provocative? | 0.71 | 2. Often deliberately does things to annoy others | 0.55 | 2. Teases a lot | 0.74 |  |  |
| 3. Is s/he easily offended, or disturbed by others? | 0.61 | 3. S/he is accepted by other children (R) | 0.37 | 3. Stubborn, sullen, or irritable | 0.67 |  |  |
| 4. Has s/he ever deliberately been physically cruel to anybody? | 0.61 | 4. Uses physical force to dominate | 0.69 | 4. Cruelty, bullying, or meanness to others/Physically attacks people | 0.714/0.588 |  |  |
| 5. Does s/he often start fights? | 0.61 | 5. Often fights with other children or bullies them | 0.70 | 5. Gets in many fights | 0.66 |  |  |
| 6. Does s/he often lie or cheat? | 0.59 | 6. Often lies or cheats | 0.65 | 6. Disobedient at home | 0.73 |  |  |
| Cronbach’s alpha | 0.71 | Cronbach’s alpha | 0.63 | Cronbach’s alpha | 0.82 |  |  |
| **Age 12 Parent-reported aggression** | | | | | | | |
| CATSS | | TEDS | | NTR | | FT12 | |
| Item | FL | Item | FL | Item | FL | Item | FL |
| Not available |  | 1. Considerate of other people's feelings ( R) | 0.69 | 1. Cruelty, bullying, or meanness to others | 0.74 | 1. Excludes others | 0.60 |
|  |  | 2. Kind to younger children | 0.47 | 2. Physically attacks people | 0.69 | 2. Teases others and violent for no reason | 0.73 |
|  |  | 3. Often fights with other children and bullies them | 0.59 | 3. Gets in many fights | 0.73 | 3. When angry, might hit, push, kick, or throw something at the person | 0.76 |
|  |  | 4. Teases, makes fun of other people | 0.62 | 4. Teases a lot | 0.75 | 4. Tease smaller/weaker students | 0.59 |
|  |  | 5. Becomes angry when corrected or punished | 0.60 | 5. Threatens people | 0.55 | 5. Scold people when upset with them | 0.68 |
|  |  | Cronbach’s alpha | .544 | Cronbach’s alpha | .732 | Cronbach’s alpha | .700 |
| **Age 14-5 Twin-reported aggression** | | | | | | | |
| CATSS | | TEDS | | NTR | | FT12 | |
| Item | FL | Item | FL | Item | FL | Item | FL |
| 1. Gotten others to gang up on someone else | 0.64 | Not available |  | 1. I’m mean to other people | 0.61 | 1. I exclude others | 0.57 |
| 2. Used physical force to get others to do what you want | 0.70 |  |  | 2. I physically attack people | 0.69 | 2. tease others and violent for no reason | 0.70 |
| 3. I get very angry and often lose my temper | 0.49 |  |  | 3. I’m quick-tempered | 0.69 | 3. spread rumours when mad | 0.58 |
| 4. I fight a lot. I can make other people do what I want | 0.65 |  |  | 4. I fight a lot | 0.67 | 4. When angry, I might hit, push, kick, or throw something at the person | 0.65 |
| 5. Threatened and bullied someone | 0.62 |  |  | 5. I bully others a lot | 0.55 | 5. Tease smaller/weaker students | 0.68 |
| Cronbach’s alpha | 0.61 | Cronbach’s alpha |  | Cronbach’s alpha | 0.65 | Cronbach’s alpha | 0.63 |
| **Age 16-18 Twin-reported aggression** | | | | | | | |
| CATSS | | TEDS | | NTR | | FT12 | |
| Item | FL | Item | FL | Item | FL | Item | FL |
| 1. Thrown a temper tantrum (for example: screaming, slamming doors, throwing things when frustrated to the “breaking point”) | 0.72 | 1. I try to be nice to other people. I care about their feelings (R) | 0.54 | 1. I'm mean to other people | 0.65 | 1. I exclude others | 0.61 |
| 2. Gotten into physical fights with other people | 0.75 | 2. I fight a lot. I can make other people do what I wan | 0.74 | 2. I fight a lot | 0.69 | 2. I get angry often, and easily get involved in quarrels or fights | 0.67 |
| 3. Gotten into verbal fights or arguments with other people | 0.80 | 3. I get very angry and often lose my temper | 0.75 | 3. I often get into arguments | 0.56 | 3. When someone yells at me, I yell back | 0.67 |
| 4. Deliberately hit another person (or an animal) in anger | 0.78 |  |  | 4. I physically attack people | 0.67 | 4. When angry, I might hit a person | 0.65 |
|  |  |  |  | 5. I bully others a lot | 0.55 | 5. tease others and violent for no reason | 0.53 |
| Cronbach’s alpha | 0.76 | Cronbach’s alpha | .052 | Cronbach’s alpha | 0.61 | Cronbach’s alpha | 0.61 |

*Note*: CATSS = Childhood and Adolescent Twin Study of Sweden; TEDS = Twins Early Development Study, NTR = Netherlands Twin Register, FT12 = FinnTwin12 sample.

**Table S3.** Descriptive statistics for the harmonized measures of aggression across the four samples

| Descriptive Statistics | | | | | | b) Descriptive statistics of square-root transformed measures | | | | | | |
| --- | --- | --- | --- | --- | --- | --- | --- | --- | --- | --- | --- | --- |
| **TEDS sample** | | | | | | | | | | | | |
|  | **Age 9** | **Age 12** | **Age 14-15** | | **Age 16-18** | **Age 9** | | **Age 12** | **Age 14** | | | **Age 16** |
| N | 3415 | 2847 | Not available | | 2174 | 3404 | | 2847 | Not available | | | 2174 |
| Mean | 0.24 | 0.27 |  | | 0.30 | 0.38 | | 0.42 |  | | | 0.41 |
| St Dev | 0.27 | 0.28 |  | | 0.35 | 0.31 | | 0.32 |  | | | 0.38 |
| Skew | 1.61 | 1.30 |  | | 1.29 | 0.09 | | -0.10 |  | | | 0.14 |
| SE Skew | 0.04 | 0.05 |  | | 0.05 | 0.04 | | 0.03 |  | | | 0.03 |
| Kurt | 3.52 | 2.28 |  | | 1.57 | -1.02 | | -1.05 |  | | | -1.36 |
| SE Kurt | 0.08 | 0.09 |  | | 0.11 | 0.08 | | 0.06 |  | | | 0.07 |
| Min | 0.00 | 0.00 |  | | 0.00 | 0.00 | | 0.00 |  | | | 0.00 |
| Max | 1.83 | 2.00 |  | | 2.00 | 1.29 | | 1.34 |  | | | 1.41 |
| **NTR sample** | | | | | | | | | | | | |
|  | **Age 10** | **Age 12** | **Age 14** | | **Age 16** | | **Age 10** | **Age 12** | | **Age 14** | | **Age 16** |
| N | 9995 | 8492 | 4532 | | 3098 | | 9995 | 8492 | | 4532 | | 3098 |
| M | 0.32 | 0.07 | 0.14 | | 0.17 | | 0.48 | 0.13 | | 0.23 | | 0.28 |
| SD | 0.30 | 0.17 | 0.23 | | 0.22 | | 0.30 | 0.24 | | 0.30 | | 0.30 |
| Skew | 1.37 | 3.49 | 2.25 | | 1.73 | | -0.08 | 1.69 | | 0.82 | | 0.46 |
| SE Skew | 0.02 | 0.03 | 0.04 | | 0.04 | | 0.02 | 0.03 | | 0.04 | | 0.04 |
| Kurt | 2.43 | 16.73 | 6.99 | | 4.24 | | -0.56 | 1.98 | | -0.55 | | -1.11 |
| SE Kurt | 0.05 | 0.05 | 0.07 | | 0.09 | | 0.05 | 0.05 | | 0.07 | | 0.09 |
| Min | 0.00 | 0.00 | 0.00 | | 0.00 | | 0.00 | 0.00 | | 0.00 | | 0.00 |
| Max | 2.00 | 2.00 | 2.00 | | 1.80 | | 1.41 | 1.41 | | 1.41 | | 1.34 |
| **FT12 sample** | | | | | | | | | | | | |
|  | **Age 9** | **Age 12** | **Age 14** | | **Age 17** | | **Age 9** | **Age 12** | | **Age 14** | | **Age 17** |
| N | Not available | 2813 | 1037 | | 2114 | | Not available | 2813 | | 1037 | | 2114 |
| M |  | 0.65 | 0.36 | | 1.87 | |  | 0.74 | | 0.48 | | 1.35 |
| SD |  | 0.45 | 0.36 | | 0.64 | |  | 0.32 | | 0.35 | | 0.21 |
| Skew |  | 0.94 | 1.54 | | 3.78 | |  | -0.49 | | -0.03 | | 1.40 |
| SE Skew |  | 0.05 | 0.08 | | 0.05 | |  | 0.05 | | 0.08 | | 0.05 |
| Kurt |  | 1.09 | 3.82 | | 43.32 | |  | 0.45 | | -0.77 | | 8.03 |
| SE Kurt |  | 0.09 | 0.15 | | 0.11 | |  | 0.09 | | 0.15 | | 0.11 |
| Min |  | 0.00 | 0.00 | | 1.00 | |  | 0.00 | | 0.00 | | 1.00 |
| Max |  | 3.00 | 2.60 | | 12.20 | |  | 1.73 | | 1.61 | | 3.49 |
| **CATSS sample** | | | | | | | | | | | | |
|  | **Age 9** | **Age 12** | | **Age 15** | **Age 18** | | **Age 9** | **Age 12** | | | **Age 15** | **Age 18** |
| N | 13500 | Not available | | 4133 | 2974 | | 13500 | Not available | | | 8239 | 2974 |
| M | 0.05 |  | | 0.76 | 2.53 | | 0.10 |  | | | 0.86 | 1.55 |
| SD | 0.11 |  | | 0.22 | 1.11 | | 0.19 |  | | | 0.12 | 0.35 |
| Skew | 3.56 |  | | 1.79 | 0.67 | | 1.69 |  | | | 0.39 | 0.24 |
| SE Skew | 0.02 |  | | 0.04 | 0.05 | | 0.02 |  | | | 0.03 | 0.05 |
| Kurt | 16.29 |  | | 5.36 | -0.05 | | 2.05 |  | | | 8.00 | -0.65 |
| SE Kurt | 0.04 |  | | 0.08 | 0.09 | | 0.04 |  | | | 0.05 | 0.09 |
| Min | 0.00 |  | | 0.00 | 1.00 | | 0.00 |  | | | 0.00 | 1.00 |
| Max | 1.00 |  | | 2.40 | 6.00 | | 1.00 |  | | | 1.61 | 2.45 |

*Note*: CATSS = Childhood and Adolescent Twin Study of Sweden; TEDS = Twins Early Development Study, NTR = Netherlands Twin Register, FT12 = FinnTwin12 sample; Min = minimum score, Max = maximum score.

**Table S4.** Descriptive statistics for all perinatal measures

| **CATTS sample** | | | | | | | | | | | |
| --- | --- | --- | --- | --- | --- | --- | --- | --- | --- | --- | --- |
|  | Maternal education | Paternal education | maternal age | paternal age | birth weight | MSDP | MSDP SQRT |  |  |  |  |
| N | 13458 | 13293 | 13239 | 13466 | 13157 | 12340 | 12340 |  |  |  |  |
| Mean | 3.870 | 3.580 | 30.930 | 33.470 | 2634.910 | 1.170 | 1.066 |  |  |  |  |
| Std. Deviation | 1.080 | 1.099 | 4.642 | 5.747 | 590.885 | 0.475 | 0.181 |  |  |  |  |
| Skew | -0.020 | 0.437 | 0.005 | 0.636 | -0.481 | 2.851 | 2.668 |  |  |  |  |
| SE Skew | 0.021 | 0.021 | 0.021 | 0.021 | 0.021 | 0.022 | 0.022 |  |  |  |  |
| Kurtosis | -1.163 | -0.096 | -0.136 | 1.020 | 0.504 | 7.216 | 5.937 |  |  |  |  |
| SE Kurtosis | 0.042 | 0.042 | 0.043 | 0.042 | 0.043 | 0.044 | 0.044 |  |  |  |  |
| Min | 1 | 1 | 14 | 15 | 422 | 1 | 1 |  |  |  |  |
| Max | 9 | 9 | 51 | 67 | 5493 | 3 | 2 |  |  |  |  |
| **NTR sample** | | | | | | | | | | | |
|  | maternal education | paternal education | maternal age | birth weight | gestation age | length breastfeed | stress pregnancy | MSDP | paternal SDP |  |  |
| N | 14295 | 14085 | 15515 | 14944 | 15065 | 12986 | 439 | 13198 | 12504 |  |  |
| Mean | 2.890 | 2.930 | 31.345 | 2522.880 | 36.631 | 2.660 | 0.551 | 1.208 | 1.499 |  |  |
| Std. Deviation | 0.850 | 0.916 | 3.962 | 542.598 | 2.521 | 1.763 | 0.937 | 0.475 | 0.777 |  |  |
| Skew | -0.256 | -0.347 | 0.032 | -0.384 | -1.096 | 0.579 | 1.573 | 2.251 | 1.137 |  |  |
| SE Skew | 0.020 | 0.021 | 0.020 | 0.020 | 0.020 | 0.021 | 0.117 | 0.021 | 0.022 |  |  |
| Kurtosis | -0.718 | -0.881 | -0.056 | 0.259 | 1.671 | -1.063 | 1.217 | 4.350 | -0.394 |  |  |
| SE Kurtosis | 0.041 | 0.041 | 0.039 | 0.040 | 0.040 | 0.043 | 0.233 | 0.043 | 0.044 |  |  |
| Min | 1 | 1 | 17.200 | 530.000 | 24.000 | 1.000 | 0.000 | 1.000 | 1.000 |  |  |
| Max | 4 | 4 | 47.38 | 4750 | 45.5 | 6 | 3 | 3 | 3 |  |  |
| **FT12 sample** | | | | | | | | | | | |
|  | maternal education | paternal education | maternal age | birth weight | gestation age | MSDP | MSDP sqrt | Paternal smoking |  |  |  |
| N | 5050 | 4498 | 5410 | 5389 | 5196 | 7153 | 7153 | 3156 |  |  |  |
| Mean | 13.240 | 12.640 | 40.925 | 2701.870 | 36.925 | 1.115 | 1.045 | 0.909 |  |  |  |
| Std. Deviation | 3.610 | 3.942 | 4.928 | 520.653 | 2.380 | 0.393 | 0.151 | 0.287 |  |  |  |
| Skew | 0.276 | 0.296 | 0.157 | -0.399 | -0.910 | 3.608 | 3.386 | -2.853 |  |  |  |
| SE Skew | 0.034 | 0.037 | 0.033 | 0.033 | 0.034 | 0.029 | 0.029 | 0.044 |  |  |  |
| Kurtosis | 0.016 | -0.343 | -0.411 | 0.541 | 2.893 | 12.635 | 10.642 | 6.146 |  |  |  |
| SE Kurtosis | 0.069 | 0.073 | 0.067 | 0.067 | 0.068 | 0.058 | 0.058 | 0.087 |  |  |  |
| Min | 7 | 7 | 28.54 | 700 | 26 | 1 | 1 | 0 |  |  |  |
| Max | 20 | 20 | 57.29 | 4530 | 48 | 3 | 1.73 | 1 |  |  |  |
| **TEDS sample** | | | | | | | | | | | |
|  | maternal education | paternal education | maternal age | paternal age | birthweight | gestation age | drink DP | length breastfeed | stress DP | low SES | MSDP |
| N | 13360 | 11942 | 13378 | 11952 | 13200 | 13448 | 13312 | 7025 | 13562 | 11872 | 13453 |
| Mean | 3.63 | 3.91 | 30.7299 | 33.2983 | 2469.8617 | 36.31 | 0.38 | 105.477 | 0.22 | 0.000483 | 0.24 |
| Std. Deviation | 1.959 | 2.215 | 4.84353 | 5.84928 | 564.28729 | 2.632 | 0.668 | 118.5489 | 0.412 | 0.999841 | 0.543 |
| Skew | 0.899 | 0.509 | -0.091 | 0.658 | -0.348 | -1.048 | 1.53 | 1.561 | 1.371 | -0.199 | 2.199 |
| SE Skew | 0.021 | 0.022 | 0.021 | 0.022 | 0.021 | 0.021 | 0.021 | 0.029 | 0.021 | 0.022 | 0.021 |
| Kurtosis | -0.143 | -0.974 | -0.035 | 1.116 | 0.218 | 1.519 | 0.908 | 1.88 | -0.12 | -0.698 | 3.75 |
| SE Kurtosis | 0.042 | 0.045 | 0.042 | 0.045 | 0.043 | 0.042 | 0.042 | 0.058 | 0.042 | 0.045 | 0.042 |
| Min | 1 | 1 | 13.92 | 15.92 | 454 | 24 | 0 | 1 | 0 | -2.6494 | 0 |
| Max | 8 | 8 | 60.22 | 65.5 | 4540 | 43 | 2 | 557 | 1 | 2.5997 | 2 |

*Note*: CATSS = Childhood and Adolescent Twin Study of Sweden; TEDS = Twins Early Development Study, NTR = Netherlands Twin Register, FT12 = FinnTwin12 sample; drink DP = drinking during pregnancy; stress DP = stress during pregnancy; length breastfeed = length of breastfeeding; low SES = low socioeconomic status; MSDP = maternal smoking during pregnancy; MSDP SQRT = MSDP square root transformed.

**Table S5.** Correlations between MSDP, perinatal measures and offspring’s aggression in the CATSS sample (a), TEDS sample (b), NTR sample (c) and FT12 sample (d).

**(a) CATSS sample**

|  | 1 | 2 | 3 | 4 | 5 | 6 | 7 | 8 |
| --- | --- | --- | --- | --- | --- | --- | --- | --- |
| 1. Agg 9 | 1 |  |  |  |  |  |  |  |
| 2. Agg 15 | .131** | 1 |  |  |  |  |  |  |
| 3. Agg 18 | .121** | .259** | 1 |  |  |  |  |  |
| 4. m education | -.070** | -.064** | .031 | 1 |  |  |  |  |
| 5. f education | -.069** | -.079** | .015 | .405** | 1 |  |  |  |
| 6. m age | -.045** | .003 | .018 | .161** | .162** | 1 |  |  |
| 7. f age | -.024** | .009 | 0.027 | .103** | .089** | .641** | 1 |  |
| 8. MSDP | .096** | .040* | .080** | -.245** | -.181** | -.022* | -.023** | 1 |

* = p < .05, ** = p< .01, Agg 9 = Aggression age 9, Agg 15 = Aggression age 15, Agg 18 = Aggression age 18, , m age = maternal age, f age = paternal age, MSDP = maternal smoking during pregnancy.

**(b) TEDS sample**

|  | 1 | 2 | 3 | 4 | 5 | 6 | 7 | 8 | 9 | 10 | 11 | 12 |
| --- | --- | --- | --- | --- | --- | --- | --- | --- | --- | --- | --- | --- |
| 1. Agg 9 | 1 |  |  |  |  |  |  |  |  |  |  |  |
| 2. Agg 12 | .374** | 1 |  |  |  |  |  |  |  |  |  |  |
| 3. Agg 16 | .205** | .188** | 1 |  |  |  |  |  |  |  |  |  |
| 4. m education | -.122** | -.023 | -.108** | 1 |  |  |  |  |  |  |  |  |
| 5. f education | -.121** | -.009 | -.104** | .547** | 1 |  |  |  |  |  |  |  |
| 6. m age | -.092** | -.031* | -.042** | .234** | .238** | 1 |  |  |  |  |  |  |
| 7. low SES | .140** | .015 | .117** | -.759** | -.779** | -.521** | 1 |  |  |  |  |  |
| 8. alcohol dp | -.008 | .011 | -0.005 | .135** | .133** | .101** | -.164** | 1 |  |  |  |  |
| 9. breastfed | -.056* | -.034* | -.069** | .217** | .182** | .162** | -.211** | .046** | 1 |  |  |  |
| 10. gest age | -.022 | .010 | .049** | .046** | .044** | .058** | -.034** | .039** | 0.02 | 1 |  |  |
| 11. stress dp | .070** | 0.014 | .025 | .009 | .001 | -.001 | -.002 | .040** | -0.005 | -.053** | 1 |  |
| 12. MSDP | .122** | .059** | .090** | -.237** | -.207** | -.123** | .264** | .049** | -.119** | -.022* | .073** | 1 |

* = p < .05, ** = p< .01, Agg 9 = Aggression age 9, Agg 12 = Aggression age 12, Agg 16 = Aggression age 16, m age = maternal age, low SES = low socio-economic status, alcohol dp = alcohol consumption during pregnancy, breastfed = length of breastfeeding, gest age = gestational age, stredd dp = stress during pregnancy, MSDP = maternal smoking during pregnancy.

**(c) NTR sample**

|  | 1 | 2 | 3 | 4 | 5 | 6 | 7 | 8 | 9 | 10 |
| --- | --- | --- | --- | --- | --- | --- | --- | --- | --- | --- |
| 1. Agg 10 | 1 |  |  |  |  |  |  |  |  |  |
| 2. Agg 12 | .510** | 1 |  |  |  |  |  |  |  |  |
| 3. Agg 14 | .241** | .229** | 1 |  |  |  |  |  |  |  |
| 4. Agg 16 | .230** | .214** | .321** | 1 |  |  |  |  |  |  |
| 5. m education | -.137** | -.114** | -.080** | -.065** | 1 |  |  |  |  |  |
| 6. f education | -.121** | -.120** | -.094** | -.048* | .454** | 1 |  |  |  |  |
| 7. m age | -.125** | -.113** | -.040** | -0.032 | .174** | .178** | 1 |  |  |  |
| 8. breastfed | -.072** | -.039** | 0.015 | 0.008 | .277** | .187** | .148** | 1 |  |  |
| 9. gest age | -0.009 | -0.001 | .031* | .050** | 0.015 | 0.013 | .054** | .020* | 1 |  |
| 10. MSDP | .115** | .096** | .088** | .058** | -.248** | -.204** | -.065** | -.157** | 0.008 | 1 |

* = p < .05, ** = p< .01, Agg 10 = Aggression age 10, Agg 12 = Aggression age 12, Agg 14 = Aggression age 14, Agg 16 = Aggression age 16, m age = maternal age, breastfed = length of breastfeeding, gest age = gestational age, stredd dp = stress during pregnancy, MSDP = maternal smoking during pregnancy.

**(d) FT12 sample**

|  | 1 | 2 | 3 | 4 | 5 | 6 | 7 | 8 |
| --- | --- | --- | --- | --- | --- | --- | --- | --- |
| 1. Agg 12 | 1 |  |  |  |  |  |  |  |
| 2. Agg 14 | .173** | 1 |  |  |  |  |  |  |
| 3. Agg 17 | .177** | .204** | 1 |  |  |  |  |  |
| 4. m education | 0.013 | -0.064 | -.083** | 1 |  |  |  |  |
| 5. f education | -0.022 | -0.016 | -.058* | .503** | 1 |  |  |  |
| 6. m age | -.110** | -0.008 | -.082** | -0.019 | -0.015 | 1 |  |  |
| 7. gest age | 0 | 0.06 | 0.035 | -0.007 | -0.011 | -0.009 | 1 |  |
| 8. MSDP | .067** | -0.011 | .050* | -.194** | -.125** | -.041* | -0.03 | 1 |

* = p < .05, ** = p< .01, Agg 12 = Aggression age 12, Agg 14 = Aggression age 14, Agg 17 = Aggression age 17, m age = maternal age, gest age = gestational age, MSDP = maternal smoking during pregnancy.

**Table S6.** Hierarchical regressions exploring the association between perinatal measures, MSDP and aggression in the TEDS sample at age 9 (a), 12 (b) and 16 (c).

(a) Criterion: Aggression age 9 TEDS sample (N = 1814, one twin randomly selected from each pair)

| Model | R | R Square | Adjusted R Square | Std. Error of the Estimate | R Square Change | F Change | df1 | df2 | Sig. F Change |
| --- | --- | --- | --- | --- | --- | --- | --- | --- | --- |
| 1 | .149a | 0.022 | 0.018 | 0.972 | 0.022 | 5.127 | 8 | 1805 | .001 |
| **2** | **.166b** | **0.027** | **0.023** | **0.969** | **0.005** | **9.751** | **1** | **1804** | **.002** |

| **Model** | **Predictors** | **B** | **Std. Error** | **Beta** | **t** | **p** |
| --- | --- | --- | --- | --- | --- | --- |
| 1 | (Constant) | 0.726 | 0.445 |  | 1.632 | 0.103 |
|  | maternal education | -0.014 | 0.019 | -0.03 | -0.74 | 0.459 |
|  | paternal education | 0.002 | 0.018 | 0.006 | 0.132 | 0.895 |
|  | maternal age | -0.007 | 0.007 | -0.033 | -1.041 | 0.298 |
|  | low ses | 0.091 | 0.067 | 0.089 | 1.367 | 0.172 |
|  | drinking during pregnancy | 0.047 | 0.034 | 0.033 | 1.405 | 0.16 |
|  | length breastfeeding | 0.000 | 0.000 | -0.033 | -1.367 | 0.172 |
|  | gestation age | -0.012 | 0.010 | -0.03 | -1.269 | 0.204 |
|  | stress during pregnancy | 0.101 | 0.057 | 0.042 | 1.78 | 0.075 |
| 2 | (Constant) | 0.668 | 0.444 |  | 1.503 | 0.133 |
|  | maternal education | -0.013 | 0.019 | -0.028 | -0.687 | 0.492 |
|  | paternal education | 0.002 | 0.018 | 0.006 | 0.13 | 0.897 |
|  | maternal age | -0.007 | 0.007 | -0.032 | -1.038 | 0.3 |
|  | low ses | 0.077 | 0.067 | 0.076 | 1.163 | 0.245 |
|  | drinking during pregnancy | 0.035 | 0.034 | 0.025 | 1.049 | 0.294 |
|  | length breastfeeding | 0.000 | 0.000 | -0.028 | -1.149 | 0.251 |
|  | gestation age | -0.012 | 0.01 | -0.028 | -1.196 | 0.232 |
|  | stress during pregnancy | 0.091 | 0.057 | 0.037 | 1.591 | 0.112 |
|  | **MSDP** | **0.194** | **0.062** | **0.075** | **3.123** | **0.002** |

(b) Criterion: Aggression age 12 TEDS sample (N = 1515, one twin randomly selected from each pair)

| Model | R | R Square | Adjusted R Square | Std. Error of the Estimate | R Square Change | F Change | df1 | df2 | Sig. F Change |
| --- | --- | --- | --- | --- | --- | --- | --- | --- | --- |
| 1 | .094 | 0.009 | 0.004 | 1.000 | 0.009 | 1.678 | 8 | 1506 | 0.099 |
| **2** | **.113** | **0.013** | **0.007** | **0.998** | **0.004** | **6.016** | **1** | **1505** | **0.014** |

| **Model** | **Predictors** | **B** | **Std. Error** | **Beta** | **t** | **p** |
| --- | --- | --- | --- | --- | --- | --- |
| 1 | (Constant) | -0.019 | 0.503 |  | -0.037 | 0.97 |
|  | maternal education | 0.011 | 0.022 | 0.023 | 0.522 | 0.602 |
|  | paternal education | 0.003 | 0.021 | 0.007 | 0.155 | 0.877 |
|  | maternal age | 0.003 | 0.008 | 0.011 | 0.331 | 0.741 |
|  | low ses | 0.046 | 0.075 | 0.045 | 0.617 | 0.537 |
|  | drinking during pregnancy | 0.081 | 0.038 | 0.056 | 2.126 | 0.034 |
|  | length breastfeeding | -0.001 | 0.000 | -0.073 | -2.717 | 0.007 |
|  | gestation age | -0.003 | 0.011 | -0.007 | -0.285 | 0.776 |
|  | stress during pregnancy | 0.04 | 0.065 | 0.016 | 0.624 | 0.533 |
| 2 | (Constant) | -0.054 | 0.503 |  | -0.108 | 0.914 |
|  | maternal education | 0.012 | 0.022 | 0.024 | 0.545 | 0.586 |
|  | paternal education | 0.003 | 0.021 | 0.007 | 0.149 | 0.881 |
|  | maternal age | 0.002 | 0.008 | 0.011 | 0.309 | 0.758 |
|  | low ses | 0.033 | 0.075 | 0.032 | 0.441 | 0.659 |
|  | drinking during pregnancy | 0.072 | 0.038 | 0.049 | 1.882 | 0.060 |
|  | length breastfeeding | -0.001 | 0.000 | -0.068 | -2.53 | 0.011 |
|  | gestation age | -0.003 | 0.011 | -0.006 | -0.245 | 0.806 |
|  | stress during pregnancy | 0.030 | 0.065 | 0.012 | 0.46 | 0.646 |
|  | **SDP** | **0.170** | **0.069** | **0.065** | **2.453** | **0.014** |

(c) Criterion: Aggression age 16 TEDS sample (N = 1206, one twin randomly selected from each pair)

| Model | R | R Square | Adjusted R Square | Std. Error of the Estimate | R Square Change | F Change | df1 | df2 | Sig. F Change |
| --- | --- | --- | --- | --- | --- | --- | --- | --- | --- |
| 1 | .160 | 0.026 | 0.019 | 0.973 | 0.026 | 3.94 | 8 | 1197 | 0.001 |
| **2** | **.175** | **0.031** | **0.023** | **0.971** | **0.005** | **6.198** | **1** | **1196** | **0.013** |

| **Model** | **Predictors** | **B** | **Std. Error** | **Beta** | **t** | **p** |
| --- | --- | --- | --- | --- | --- | --- |
| 1 | (Constant) | -0.217 | 0.546 |  | -0.398 | 0.691 |
|  | maternal education | 0.001 | 0.023 | 0.001 | 0.022 | 0.983 |
|  | paternal education | -0.015 | 0.023 | -0.035 | -0.644 | 0.520 |
|  | maternal age | 0.004 | 0.009 | 0.019 | 0.507 | 0.612 |
|  | low ses | 0.092 | 0.083 | 0.09 | 1.108 | 0.268 |
|  | drinking during pregnancy | 0.075 | 0.041 | 0.053 | 1.807 | 0.071 |
|  | length breastfeeding | -0.001 | 0.000 | -0.076 | -2.557 | 0.011 |
|  | gestation age | 0.004 | 0.012 | 0.01 | 0.337 | 0.736 |
|  | stress during pregnancy | 0.130 | 0.070 | 0.053 | 1.849 | 0.065 |
| 2 | (Constant) | -0.223 | 0.545 |  | -0.408 | 0.683 |
|  | maternal education | 0.001 | 0.023 | 0.002 | 0.031 | 0.975 |
|  | paternal education | -0.015 | 0.023 | -0.035 | -0.652 | 0.514 |
|  | maternal age | 0.004 | 0.009 | 0.018 | 0.469 | 0.639 |
|  | low ses | 0.077 | 0.083 | 0.076 | 0.931 | 0.352 |
|  | drinking during pregnancy | 0.064 | 0.042 | 0.045 | 1.539 | 0.124 |
|  | length breastfeeding | -0.001 | 0.000 | -0.071 | -2.402 | 0.016 |
|  | gestation age | 0.004 | 0.012 | 0.009 | 0.320 | 0.749 |
|  | stress during pregnancy | 0.120 | 0.070 | 0.049 | 1.711 | 0.087 |
|  | **MSDP** | **0.201** | **0.081** | **0.073** | **2.490** | **0.013** |

**Table S7.** Hierarchical regressions exploring the association between perinatal measures, MSDP and aggression in the NTR sample at age 10 (a), 12 (b), 14 (c) and 16 (d).

(a) Criterion: Aggression age 10 NTR sample (N = 7,061, one twin randomly selected from each pair)

|  | R | R Square | Adjusted R Square | Std. Error of the Estimate | R Square Change | F Change | df1 | df2 | Sig. F Change |
| --- | --- | --- | --- | --- | --- | --- | --- | --- | --- |
| 1 | .168 | 0.028 | 0.028 | 0.975 | 0.028 | 41.215 | 5 | 7055 | .001 |
| **2** | **.183** | **0.033** | **0.033** | **0.972** | **0.005** | **37.108** | **1** | **7054** | **.001** |

| **Model** | **Predictors** | **B** | **Std. Error** | **Beta** | **t** | **p** |
| --- | --- | --- | --- | --- | --- | --- |
| 1 | (Constant) | 1.235 | 0.193 |  | 6.387 | 0.000 |
|  | maternal education | -0.101 | 0.016 | -0.085 | -6.335 | 0.000 |
|  | paternal education | -0.061 | 0.014 | -0.056 | -4.256 | 0.000 |
|  | maternal age | -0.021 | 0.003 | -0.083 | -6.876 | 0.000 |
|  | breastfeeding | -0.013 | 0.007 | -0.024 | -1.917 | 0.055 |
|  | gestation age | -0.002 | 0.005 | -0.005 | -0.452 | 0.651 |
| 2 | (Constant) | 1.006 | 0.197 |  | 5.118 | 0.000 |
|  | maternal education | -0.085 | 0.016 | -0.072 | -5.316 | 0.000 |
|  | paternal education | -0.052 | 0.014 | -0.048 | -3.661 | 0.000 |
|  | maternal age | -0.021 | 0.003 | -0.083 | -6.923 | 0.000 |
|  | breastfeeding | -0.01 | 0.007 | -0.017 | -1.363 | 0.173 |
|  | gestation age | -0.003 | 0.005 | -0.007 | -0.619 | 0.536 |
|  | **MSDP** | **0.149** | **0.024** | **0.074** | **6.092** | **0.000** |

(b) Criterion: Aggression age 12 NTR sample (N = 5,865, one twin randomly selected from each pair)

|  | R | R Square | Adjusted R Square | Std. Error of the Estimate | R Square Change | F Change | df1 | df2 | Sig. F Change |
| --- | --- | --- | --- | --- | --- | --- | --- | --- | --- |
| 1 | .141 | 0.02 | 0.019 | 0.952 | 0.02 | 23.974 | 5 | 5869 | 0.001 |
| **2** | **.157** | **0.025** | **0.024** | **0.950** | **0.005** | **27.223** | **1** | **5868** | **0.001** |

| **Model** | **Predictors** | **B** | **Std. Error** | **Beta** | **t** | **p** |
| --- | --- | --- | --- | --- | --- | --- |
| 1 | (Constant) | 0.976 | 0.208 |  | 4.688 | 0.000 |
|  | maternal education | -0.054 | 0.017 | -0.047 | -3.175 | 0.002 |
|  | paternal education | -0.077 | 0.015 | -0.073 | -5.014 | 0.000 |
|  | maternal age | -0.021 | 0.003 | -0.081 | -6.109 | 0.000 |
|  | breastfeeding | 0.003 | 0.008 | 0.006 | 0.417 | 0.676 |
|  | gestation age | 0.000 | 0.005 | 0.000 | -0.007 | 0.995 |
| 2 | (Constant) | 0.765 | 0.212 |  | 3.614 | 0.000 |
|  | maternal education | -0.041 | 0.017 | -0.036 | -2.403 | 0.016 |
|  | paternal education | -0.069 | 0.015 | -0.065 | -4.454 | 0.000 |
|  | maternal age | -0.021 | 0.003 | -0.083 | -6.257 | 0.000 |
|  | breastfeeding | 0.007 | 0.008 | 0.012 | 0.883 | 0.377 |
|  | gestation age | 0.000 | 0.005 | -0.001 | -0.079 | 0.937 |
|  | **MSDP** | **0.138** | **0.027** | **0.070** | **5.218** | **0.000** |

(c) Criterion: Aggression age 14 NTR sample (N = 3,807, one twin randomly selected from each pair)

| Model | R | R Square | Adjusted R Square | Std. Error of the Estimate | R Square Change | F Change | df1 | df2 | Sig. F Change |
| --- | --- | --- | --- | --- | --- | --- | --- | --- | --- |
| 1 | .125 | 0.016 | 0.014 | 0.983 | 0.016 | 12.143 | 5 | 3801 | 0.000 |
| **2** | **.138** | **0.019** | **0.017** | **0.982** | **0.003** | **12.372** | **1** | **3800** | **0.000** |

| **Model** | **Predictors** | **B** | **Std. Error** | **Beta** | **t** | **p** |
| --- | --- | --- | --- | --- | --- | --- |
| 1 | (Constant) | 0.086 | 0.270 |  | 0.320 | 0.749 |
|  | maternal education | -0.067 | 0.022 | -0.056 | -3.040 | 0.002 |
|  | paternal education | -0.084 | 0.020 | -0.076 | -4.220 | 0.000 |
|  | maternal age | -0.009 | 0.004 | -0.035 | -2.108 | 0.035 |
|  | breastfeeding | 0.026 | 0.010 | 0.045 | 2.705 | 0.007 |
|  | gestation age | 0.015 | 0.007 | 0.038 | 2.338 | 0.019 |
| 2 | (Constant) | -0.088 | 0.274 |  | -0.323 | 0.747 |
|  | maternal education | -0.056 | 0.022 | -0.046 | -2.498 | 0.013 |
|  | paternal education | -0.077 | 0.020 | -0.070 | -3.841 | 0.000 |
|  | maternal age | -0.009 | 0.004 | -0.036 | -2.185 | 0.029 |
|  | breastfeeding | 0.029 | 0.010 | 0.050 | 2.994 | 0.003 |
|  | gestation age | 0.015 | 0.007 | 0.036 | 2.223 | 0.026 |
|  | **MSDP** | **0.125** | **0.036** | **0.059** | **3.517** | **0.000** |

(d) Criterion: Aggression age 16 NTR sample (N = 2,073, one twin randomly selected from each pair)

| Model | R | R Square | Adjusted R Square | Std. Error of the Estimate | R Square Change | F Change | df1 | df2 | Sig. F Change |
| --- | --- | --- | --- | --- | --- | --- | --- | --- | --- |
| 1 | .068 | 0.005 | 0.002 | 0.973 | 0.005 | 1.903 | 5 | 2066 | 0.091 |
| **2** | **.081** | **0.007** | **0.004** | **0.972** | **0.002** | **4.162** | **1** | **2065** | **0.041** |

| **Model** | **Predictors** | **B** | **Std. Error** | **Beta** | **t** | **p** |
| --- | --- | --- | --- | --- | --- | --- |
| 1 | (Constant) | -0.258 | 0.357 |  | -0.723 | 0.470 |
|  | maternal education | -0.060 | 0.030 | -0.051 | -2.024 | 0.043 |
|  | paternal education | -0.024 | 0.027 | -0.022 | -0.910 | 0.363 |
|  | maternal age | 0.001 | 0.006 | 0.002 | 0.107 | 0.915 |
|  | breastfeeding | 0.005 | 0.013 | 0.010 | 0.419 | 0.675 |
|  | gestation age | 0.011 | 0.009 | 0.028 | 1.249 | 0.212 |
| 2 | (Constant) | -0.399 | 0.364 |  | -1.099 | 0.272 |
|  | maternal education | -0.052 | 0.030 | -0.044 | -1.731 | 0.084 |
|  | paternal education | -0.020 | 0.027 | -0.018 | -0.736 | 0.462 |
|  | maternal age | 0.000 | 0.006 | 0.001 | 0.057 | 0.954 |
|  | breastfeeding | 0.009 | 0.013 | 0.016 | 0.681 | 0.496 |
|  | gestation age | 0.011 | 0.009 | 0.027 | 1.213 | 0.225 |
|  | **MSDP** | **0.096** | **0.047** | **0.046** | **2.040** | **0.041** |

**Table S8.** Hierarchical regressions exploring the association between perinatal measures, MSDP and aggression in the FT12 sample at age 12 (a), 14 (b), and 17 (c).

1. Criterion: Aggression age 12 FT12 sample (N = 2261, one twin randomly selected from each pair)

| Model | R | R Square | Adjusted R Square | Std. Error of the Estimate | R Square Change | F Change | df1 | df2 | Sig. F Change |
| --- | --- | --- | --- | --- | --- | --- | --- | --- | --- |
| 1 | .108 | 0.012 | 0.01 | 0.310 | 0.012 | 6.701 | 4 | 2256 | 0.001 |
| **2** | **.139** | **0.019** | **0.017** | **0.309** | **0.008** | **17.494** | **1** | **2255** | **0.001** |

| **Model** | **Predictors** | **B** | **Std. Error** | **Beta** | **t** | **p** |
| --- | --- | --- | --- | --- | --- | --- |
| 1 | (Constant) | 1.078 | 0.120 |  | 8.956 | 0.000 |
|  | maternal education | -0.007 | 0.001 | -0.108 | -5.136 | 0.000 |
|  | paternal education | 0.001 | 0.002 | 0.006 | 0.267 | 0.789 |
|  | maternal age | -0.001 | 0.002 | -0.008 | -0.313 | 0.755 |
|  | gestation age | -0.002 | 0.003 | -0.012 | -0.590 | 0.555 |
| 2 | (Constant) | 0.854 | 0.131 |  | 6.500 | 0.000 |
|  | maternal education | -0.007 | 0.001 | -0.104 | -4.987 | 0.000 |
|  | paternal education | 0.001 | 0.002 | 0.009 | 0.360 | 0.719 |
|  | maternal age | 0.001 | 0.002 | 0.009 | 0.363 | 0.717 |
|  | gestation age | -0.001 | 0.003 | -0.009 | -0.453 | 0.650 |
|  | **MSDP** | **0.170** | **0.041** | **0.089** | **4.183** | **0.000** |

(b) Criterion: Aggression age 14 FT12 sample (N = 848, one twin randomly selected from each pair)

| Model | R | R Square | Adjusted R Square | St Error | R Square Change | F Change | df1 | df2 | Sig. F Change |
| --- | --- | --- | --- | --- | --- | --- | --- | --- | --- |
| 1 | .080 | 0.006 | 0.002 | 1.003 | 0.006 | 1.366 | 4 | 843 | 0.244 |
| **2** | **.082** | **0.007** | **0.001** | **1.000** | **0.001** | **0.188** | **1** | **842** | **0.665** |

| **Model** | **Predictors** | **B** | **Std. Error** | **Beta** | **t** | **p** |
| --- | --- | --- | --- | --- | --- | --- |
| 1 | (Constant) | -0.969 | 0.684 |  | -1.418 | 0.157 |
|  | maternal education | 0.006 | 0.007 | 0.028 | 0.803 | 0.422 |
|  | paternal education | -0.009 | 0.011 | -0.034 | -0.843 | 0.399 |
|  | maternal age | -0.008 | 0.012 | -0.026 | -0.642 | 0.521 |
|  | gestation age | 0.025 | 0.016 | 0.055 | 1.596 | 0.111 |
| 2 | (Constant) | -1.088 | 0.736 |  | -1.477 | 0.140 |
|  | maternal education | 0.006 | 0.007 | 0.028 | 0.805 | 0.421 |
|  | paternal education | -0.009 | 0.011 | -0.033 | -0.817 | 0.414 |
|  | maternal age | -0.007 | 0.012 | -0.023 | -0.558 | 0.577 |
|  | gestation age | 0.025 | 0.016 | 0.056 | 1.612 | 0.107 |
|  | **MSDP** | **0.086** | **0.197** | **0.015** | **0.434** | **0.665** |

(c) Criterion: Aggression age 17 FT12 sample (N = 1,764, one twin randomly selected from each pair)

| Model | R | R Square | Adjusted R Square | Std. Error of the Estimate | R Square Change | F Change | df1 | df2 | Sig. F Change |
| --- | --- | --- | --- | --- | --- | --- | --- | --- | --- |
| 1 | .100 | 0.010 | 0.008 | 0.982 | 0.010 | 4.405 | 4 | 1759 | 0.002 |
| **2** | **.113** | **0.013** | **0.010** | **0.981** | **0.003** | **5.216** | **1** | **1758** | **0.023** |

| **Model** | **Predictors** | **B** | **Std. Error** | **Beta** | **t** | **p** |
| --- | --- | --- | --- | --- | --- | --- |
| 1 | (Constant) | 0.241 | 0.437 |  | 0.552 | 0.581 |
|  | maternal education | -0.010 | 0.005 | -0.047 | -1.976 | 0.048 |
|  | paternal education | -0.012 | 0.007 | -0.050 | -1.831 | 0.067 |
|  | maternal age | -0.013 | 0.008 | -0.046 | -1.665 | 0.096 |
|  | gestation age | 0.013 | 0.010 | 0.030 | 1.253 | 0.211 |
| 2 | (Constant) | -0.202 | 0.478 |  | -0.423 | 0.673 |
|  | maternal education | -0.010 | 0.005 | -0.047 | -1.987 | 0.047 |
|  | paternal education | -0.012 | 0.007 | -0.048 | -1.747 | 0.081 |
|  | maternal age | -0.010 | 0.008 | -0.037 | -1.333 | 0.183 |
|  | gestation age | 0.014 | 0.010 | 0.032 | 1.369 | 0.171 |
|  | **MSDP** | **0.344** | **0.151** | **0.055** | **2.284** | **0.023** |

**Table S9.** Hierarchical regressions exploring the association between perinatal measures, MSDP and aggression in the CATSS sample at age 10 (a), 15 (b), and 18 (c).

(a) Criterion: Aggression age 9 CATSS sample (N = 12,078, one twin randomly selected from each pair)

| Model | R | R Square | Adjusted R Square | Std. Error of the Estimate | R Square Change | F Change | df1 | df2 | Sig. F Change |
| --- | --- | --- | --- | --- | --- | --- | --- | --- | --- |
| 1 | .088 | 0.008 | 0.007 | 1.018 | 0.008 | 23.622 | 4 | 12073 | 0.000 |
| **2** | **.117** | **0.014** | **0.013** | **1.015** | **0.006** | **72.232** | **1** | **12072** | **0.000** |

| **Model** | **Predictor** | **B** | **SE** | **Beta** | **t** | **p** |
| --- | --- | --- | --- | --- | --- | --- |
| 1 | Constant | 0.550 | 0.071 |  | 7.771 | 0.000 |
|  | maternal education | -0.046 | 0.009 | -0.048 | -4.857 | 0.000 |
|  | paternal education | -0.041 | 0.009 | -0.044 | -4.382 | 0.000 |
|  | maternal age | -0.008 | 0.003 | -0.035 | -2.892 | 0.004 |
|  | paternal age | 0.001 | 0.002 | 0.008 | 0.675 | 0.500 |
| 2 | (Constant) | -0.008 | 0.096 |  | -0.081 | 0.935 |
|  | maternal education | -0.030 | 0.010 | -0.032 | -3.167 | 0.002 |
|  | paternal education | -0.033 | 0.009 | -0.036 | -3.555 | 0.000 |
|  | maternal age | -0.008 | 0.003 | -0.038 | -3.163 | 0.002 |
|  | paternal age | 0.002 | 0.002 | 0.010 | 0.815 | 0.415 |
|  | **MSDP** | **0.453** | **0.053** | **0.080** | **8.499** | **0.000** |

(b) Criterion: Aggression age 15 CATSS sample (N = 7,181, one twin randomly selected from each pair)

| Model | R | R Square | Adjusted R Square | Std. Error of the Estimate | R Square Change | F Change | df1 | df2 | Sig. F Change |
| --- | --- | --- | --- | --- | --- | --- | --- | --- | --- |
| 1 | .089 | 0.008 | 0.007 | 0.9921161 | 0.008 | 14.414 | 4 | 7176 | 0.000 |
| **2** | **.094** | **0.009** | **0.008** | **0.99171672** | **0.001** | **6.781** | **1** | **7175** | **0.009** |

| **Model** | **Predictor** | **B** | **Std. Error** | **Beta** | **t** | **p** |
| --- | --- | --- | --- | --- | --- | --- |
| 1 | (Constant) | 0.248 | 0.091 |  | 2.720 | 0.007 |
|  | maternal education | -0.024 | 0.012 | -0.026 | -2.057 | 0.040 |
|  | paternal education | -0.068 | 0.012 | -0.076 | -5.893 | 0.000 |
|  | maternal age | 0.000 | 0.004 | 0.002 | 0.136 | 0.892 |
|  | paternal age | 0.002 | 0.003 | 0.013 | 0.809 | 0.419 |
| 2 | (Constant) | 0.032 | 0.123 |  | 0.260 | 0.795 |
|  | maternal education | -0.019 | 0.012 | -0.021 | -1.578 | 0.115 |
|  | paternal education | -0.067 | 0.012 | -0.074 | -5.745 | 0.000 |
|  | maternal age | 0.000 | 0.004 | 0.001 | 0.090 | 0.928 |
|  | paternal age | 0.002 | 0.003 | 0.013 | 0.849 | 0.396 |
|  | **MSDP** | **0.179** | **0.069** | **0.031** | **2.604** | **0.009** |

(c) Criterion: Aggression age 18 CATSS sample (N = 2,634, one twin randomly selected from each pair)

| Model | R | R Square | Adjusted R Square | Std. Error of the Estimate | R Square Change | F Change | df1 | df2 | Sig. F Change |
| --- | --- | --- | --- | --- | --- | --- | --- | --- | --- |
| 1 | .060 | 0.004 | 0.002 | 1.001 | 0.004 | 2.404 | 4 | 2629 | 0.048 |
| **2** | **.129** | **0.017** | **0.015** | **0.995** | **0.013** | **34.556** | **1** | **2628** | **0.000** |

| **Model** | **Predictors** | **B** | **Std. Error** | **Beta** | **t** | **p** |
| --- | --- | --- | --- | --- | --- | --- |
| 1 | (Constant) | -0.295 | 0.148 |  | -1.997 | 0.046 |
|  | maternal education | 0.045 | 0.020 | 0.049 | 2.297 | 0.022 |
|  | paternal education | 0.012 | 0.019 | 0.014 | 0.635 | 0.525 |
|  | maternal age | -0.004 | 0.006 | -0.019 | -0.698 | 0.485 |
|  | paternal age | 0.006 | 0.005 | 0.033 | 1.235 | 0.217 |
| 2 | (Constant) | -1.050 | 0.195 |  | -5.381 | 0.000 |
|  | maternal education | 0.065 | 0.020 | 0.070 | 3.294 | 0.001 |
|  | paternal education | 0.020 | 0.019 | 0.022 | 1.027 | 0.305 |
|  | maternal age | -0.005 | 0.006 | -0.023 | -0.880 | 0.379 |
|  | paternal age | 0.006 | 0.005 | 0.035 | 1.316 | 0.188 |
|  | **MSDP** | **0.624** | **0.106** | **0.117** | **5.878** | **0.000** |

**Table S10.** Hierarchical regressions exploring the effect of heavy MSDP on offspring’s aggressive behaviour in the TEDS (a), NTR (b) FT12 (c), and CATSS (d) samples.

**(a) TEDS sample**

Criterion = Aggression Age 9 TEDS

| Model | R | R Square | Adjusted R Square | Std. Error | R Square Change | F Change | Sig. F Change |
| --- | --- | --- | --- | --- | --- | --- | --- |
| 1 | 0.120 | 0.014 | 0.014 | 0.983 | 0.014 | 49.196 | 0.000 |
| 2 | 0.123 | 0.015 | 0.015 | 0.983 | 0.001 | 2.347 | 0.126 |

*Note*: Model 1 = explored the role of MSDP, Model 2 = explored the effect of heavy MSDP above and beyond MSDP irrespective of quantity; 1 twin randomly selected out of every pair.

| Model | Predictors | B | Std. Error | Beta | t | p |
| --- | --- | --- | --- | --- | --- | --- |
| Model 1 | (Constant) | -0.048 | 0.018 |  | -2.634 | 0.008 |
|  | MSDP Y/N | 0.341 | 0.049 | 0.120 | 7.014 | 0.000 |
| Model 2 | (Constant) | -0.048 | 0.018 |  | -2.635 | 0.008 |
|  | MSDP Y/N | 0.299 | 0.056 | 0.105 | 5.351 | 0.000 |
|  | heavy MSDP Y/N | 0.155 | 0.101 | 0.030 | 1.532 | 0.126 |

Criterion = Aggression Age 12 TEDS

| Model | R | R Square | Adjusted R Square | Std. Error | R Square Change | F Change | Sig. F Change |
| --- | --- | --- | --- | --- | --- | --- | --- |
| 1 | 0.055 | 0.003 | 0.003 | 0.317 | 0.003 | 17.171 | 0.000 |
| 2 | 0.058 | 0.003 | 0.003 | 0.317 | 0.000 | 2.467 | 0.116 |

*Note*: Model 1 = explored the role of MSDP, Model 2 = explored the effect of heavy MSDP above and beyond MSDP irrespective of quantity; 1 twin randomly selected out of every pair.

| Model | Predictors | B | Std. Error | Beta | t | p |
| --- | --- | --- | --- | --- | --- | --- |
| 1 | (Constant) | 0.417 | 0.004 |  | 92.590 | 0.000 |
|  | MSDP Y/N | 0.050 | 0.012 | 0.055 | 4.144 | 0.000 |
| 2 | (Constant) | 0.417 | 0.004 |  | 92.602 | 0.000 |
|  | MSDP Y/N | 0.040 | 0.014 | 0.043 | 2.811 | 0.005 |
|  | heavy MSDP Y/N | 0.040 | 0.025 | 0.024 | 1.571 | 0.116 |

Criterion = Aggression Age 16 TEDS

| Model | R | R Square | Adjusted R Square | Std. Error of the Estimate | R Square Change | F Change | Sig. F Change |
| --- | --- | --- | --- | --- | --- | --- | --- |
| 1 | 0.089 | 0.008 | 0.008 | 0.988 | 0.008 | 40.104 | 0.000 |
| 2 | 0.091 | 0.008 | 0.008 | 0.988 | 0.000 | 1.621 | 0.203 |

*Note*: Model 1 = explored the role of MSDP, Model 2 = explored the effect of heavy MSDP above and beyond MSDP irrespective of quantity; 1 twin randomly selected out of every pair.

| Model | Predictors | B | Std. Error | Beta | t | p |
| --- | --- | --- | --- | --- | --- | --- |
| 1 | (Constant) | -0.031 | 0.015 |  | -2.100 | 0.036 |
|  | MSDP Y/N | 0.268 | 0.042 | 0.089 | 6.333 | 0.000 |
| 2 | (Constant) | -0.031 | 0.015 |  | -2.100 | 0.036 |
|  | MSDP Y/N | 0.237 | 0.049 | 0.079 | 4.859 | 0.000 |
|  | heavy MSDP Y/N | 0.113 | 0.089 | 0.021 | 1.273 | 0.203 |

**(b) NTR sample**

Criterion = Aggression age 10

|  | R | R Square | Adjusted R Square | Std. Error | R Square Change | F Change | Sig. F Change |
| --- | --- | --- | --- | --- | --- | --- | --- |
| 1 | 0.103 | 0.011 | 0.010 | 0.993 | 0.011 | 86.680 | 0.000 |
| 2 | 0.116 | 0.013 | 0.013 | 0.992 | 0.003 | 22.921 | 0.000 |

*Note*: Model 1 = explored the role of MSDP, Model 2 = explored the effect of heavy MSDP above and beyond MSDP irrespective of quantity; 1 twin randomly selected out of every pair.

| Model | Predictors | B | Std. Error | Beta | t | p |
| --- | --- | --- | --- | --- | --- | --- |
| 1 | (Constant) | -0.057 | 0.012 |  | -4.627 | 0.000 |
|  | MSDP Y/N | 0.262 | 0.028 | 0.103 | 9.310 | 0.000 |
| 2 | (Constant) | -0.057 | 0.012 |  | -4.633 | 0.000 |
|  | MSDP Y/N | 0.204 | 0.031 | 0.080 | 6.645 | 0.000 |
|  | heavy MSDP Y/N | 0.310 | 0.065 | 0.058 | 4.788 | 0.000 |

Criterion = Aggression age 12

|  | R | R Square | Adjusted R Square | Std. Error | R Square Change | F Change | Sig. F Change |
| --- | --- | --- | --- | --- | --- | --- | --- |
| 1 | 0.086 | 0.007 | 0.007 | 0.973 | 0.007 | 51.306 | 0.000 |
| 2 | 0.096 | 0.009 | 0.009 | 0.972 | 0.002 | 12.385 | 0.000 |

*Note*: Model 1 = explored the role of MSDP, Model 2 = explored the effect of heavy MSDP above and beyond MSDP irrespective of quantity; 1 twin randomly selected out of every pair.

| Model | Predictors | B | Std. Error | Beta | t | p |
| --- | --- | --- | --- | --- | --- | --- |
| 1 | (Constant) | -0.077 | 0.013 |  | -5.909 | 0.000 |
|  | MSDP Y/N | 0.219 | 0.031 | 0.086 | 7.163 | 0.000 |
| 2 | (Constant) | -0.077 | 0.013 |  | -5.914 | 0.000 |
|  | MSDP Y/N | 0.173 | 0.033 | 0.068 | 5.209 | 0.000 |
|  | heavy MSDP Y/N | 0.251 | 0.071 | 0.046 | 3.519 | 0.000 |

Criterion = Aggression age 14

| Model | R | R Square | Adjusted R Square | Std. error | R Square Change | F Change | Sig. F Change |
| --- | --- | --- | --- | --- | --- | --- | --- |
| 1 | 0.079 | 0.006 | 0.006 | 0.997 | 0.006 | 28.006 | 0.000 |
| 2 | 0.088 | 0.008 | 0.007 | 0.996 | 0.002 | 7.267 | 0.007 |

*Note*: Model 1 = explored the role of MSDP, Model 2 = explored the effect of heavy MSDP above and beyond MSDP irrespective of quantity; 1 twin randomly selected out of every pair.

| Model | Predictors | B | Std. Error | Beta | t | p |
| --- | --- | --- | --- | --- | --- | --- |
| 1 | (Constant) | -0.037 | 0.016 |  | -2.260 | 0.024 |
|  | MSDP Y/N | 0.207 | 0.039 | 0.079 | 5.292 | 0.000 |
| 2 | (Constant) | -0.037 | 0.016 |  | -2.261 | 0.024 |
|  | MSDP Y/N | 0.165 | 0.042 | 0.063 | 3.942 | 0.000 |
|  | heavy MSDP Y/N | 0.262 | 0.097 | 0.043 | 2.696 | 0.007 |

Criterion = Aggression age 16

| Model | R | R Square | Adjusted R Square | Std. Error | R Square Change | F Change | Sig. F Change |
| --- | --- | --- | --- | --- | --- | --- | --- |
| 1 | 0.068 | 0.005 | 0.004 | 0.984 | 0.005 | 11.524 | 0.001 |
| 2 | 0.070 | 0.005 | 0.004 | 0.984 | 0.000 | 0.746 | 0.388 |

*Note*: Model 1 = explored the role of MSDP, Model 2 = explored the effect of heavy MSDP above and beyond MSDP irrespective of quantity; 1 twin randomly selected out of every pair.

| Model | Predictors | B | Std. Error | Beta | t | p |
| --- | --- | --- | --- | --- | --- | --- |
| 1 | (Constant) | -0.089 | 0.022 |  | -4.121 | 0.000 |
|  | MSDP Y/N | 0.173 | 0.051 | 0.068 | 3.395 | 0.001 |
| 2 | (Constant) | -0.089 | 0.022 |  | -4.121 | 0.000 |
|  | MSDP Y/N | 0.192 | 0.055 | 0.075 | 3.464 | 0.001 |
|  | heavy MSDP Y/N | -0.104 | 0.121 | -0.019 | -0.864 | 0.388 |

**(c) FT12 sample**

Criterion = Aggression age 12

| Model | R | R Square | Adjusted R Square | Std. Error of | R Square Change | F Change | Sig. F Change |
| --- | --- | --- | --- | --- | --- | --- | --- |
| 1 | 0.083 | 0.007 | 0.006 | 0.320 | 0.007 | 16.056 | 0.000 |
| 2 | 0.083 | 0.007 | 0.006 | 0.320 | 0.000 | 0.092 | 0.761 |

*Note*: Model 1 = explored the role of MSDP, Model 2 = explored the effect of heavy MSDP above and beyond MSDP irrespective of quantity; 1 twin randomly selected out of every pair.

| Model | Predictors | B | Std. Error | Beta | t | p |
| --- | --- | --- | --- | --- | --- | --- |
| 1 | (Constant) | 0.728 | 0.007 |  | 102.658 | 0.000 |
|  | MSDP Y/N | 0.081 | 0.020 | 0.083 | 4.007 | 0.000 |
| 2 | (Constant) | 0.728 | 0.007 |  | 102.629 | 0.000 |
|  | MSDP Y/N | 0.078 | 0.024 | 0.079 | 3.284 | 0.001 |
|  | heavy MSDP Y/N | 0.012 | 0.040 | 0.007 | 0.304 | 0.761 |

Criterion = Aggression age 14

| Model | R | R Square | Adjusted R Square | Std. Error | R Square Change | F Change | Sig. F Change |
| --- | --- | --- | --- | --- | --- | --- | --- |
| 1 | 0.023 | 0.001 | -0.001 | 1.023 | 0.001 | 0.451 | 0.502 |
| 2 | 0.050 | 0.002 | 0.000 | 1.023 | 0.002 | 1.693 | 0.194 |

*Note*: Model 1 = explored the role of MSDP, Model 2 = explored the effect of heavy MSDP above and beyond MSDP irrespective of quantity; 1 twin randomly selected out of every pair.

| Model | Predictors | B | Std. Error | Beta | t | p |
| --- | --- | --- | --- | --- | --- | --- |
| 1 | (Constant) | 0.045 | 0.038 |  | 1.209 | 0.227 |
|  | MSDP Y/N | -0.066 | 0.099 | -0.023 | -0.671 | 0.502 |
| 2 | (Constant) | 0.044 | 0.038 |  | 1.182 | 0.238 |
|  | MSDP Y/N | -0.134 | 0.112 | -0.046 | -1.200 | 0.230 |
|  | heavy MSDP Y/N | 0.253 | 0.194 | 0.050 | 1.301 | 0.194 |

Criterion = Aggression age 17

| Model | R | R Square | Adjusted R Square | Std. Error | R Square Change | F Change | Sig. F Change |
| --- | --- | --- | --- | --- | --- | --- | --- |
| 1 | 0.044 | 0.002 | 0.001 | 1.012 | 0.002 | 3.819 | 0.050 |
| 2 | 0.050 | 0.003 | 0.002 | 1.012 | 0.001 | 1.247 | 0.264 |

*Note*: Model 1 = explored the role of MSDP, Model 2 = explored the effect of heavy MSDP above and beyond MSDP irrespective of quantity; 1 twin randomly selected out of every pair.

| Model | Predictors | B | Std. Error | Beta | t | p |
| --- | --- | --- | --- | --- | --- | --- |
| 1 | (Constant) | 0.015 | 0.024 |  | 0.608 | 0.543 |
|  | MSDP Y/N | 0.138 | 0.071 | 0.044 | 1.954 | 0.051 |
| 2 | (Constant) | 0.014 | 0.024 |  | 0.593 | 0.553 |
|  | MSDP Y/N | 0.091 | 0.082 | 0.029 | 1.104 | 0.27 |
|  | heavy MSDP Y/N | 0.155 | 0.139 | 0.029 | 1.117 | 0.264 |

**(d) CATSS sample**

Criterion = Aggression age 9

| *Model* | *R* | *R Square* | *Adjusted R Square* | *Std. Error* | *R Square Change* | *F Change* | *Sig. F Change* |
| --- | --- | --- | --- | --- | --- | --- | --- |
| 1 | 0.088 | 0.008 | 0.008 | 1.022 | 0.008 | 96.222 | 0.000 |
| 2 | 0.096 | 0.009 | 0.009 | 1.021 | 0.002 | 18.709 | 0.000 |

*Note*: Model 1 = explored the role of MSDP, Model 2 = explored the effect of heavy MSDP above and beyond MSDP irrespective of quantity; 1 twin randomly selected out of every pair.

| Model | Predictors | B | Std. Error | Beta | t | p |
| --- | --- | --- | --- | --- | --- | --- |
| 1 | (Constant) | 0.006 | 0.010 |  | 0.599 | 0.549 |
|  | MSDP Y/N | 0.272 | 0.028 | 0.088 | 9.809 | 0.000 |
| 2 | (Constant) | 0.006 | 0.010 |  | 0.600 | 0.549 |
|  | MSDP Y/N | 0.192 | 0.033 | 0.062 | 5.757 | 0.000 |
|  | heavy MSDP Y/N | 0.237 | 0.055 | 0.047 | 4.325 | 0.000 |

Criterion = Aggression age 15

| Model | R | R Square | Adjusted R Square | Std. Error | R Square Change | F Change | Sig. F Change |
| --- | --- | --- | --- | --- | --- | --- | --- |
| 1 | 0.036 | 0.001 | 0.001 | 1.013 | 0.001 | 4.137 | 0.042 |
| 2 | 0.041 | 0.002 | 0.001 | 1.013 | 0.000 | 1.216 | 0.270 |

*Note*: Model 1 = explored the role of MSDP, Model 2 = explored the effect of heavy MSDP above and beyond MSDP irrespective of quantity; 1 twin randomly selected out of every pair.

| Model | Predictors | B | Std. Error | Beta | t | p |
| --- | --- | --- | --- | --- | --- | --- |
| 1 | (Constant) | -0.011 | 0.019 |  | -0.584 | 0.559 |
|  | MSDP Y/N | 0.113 | 0.055 | 0.036 | 2.034 | 0.042 |
| 2 | (Constant) | -0.011 | 0.019 |  | -0.584 | 0.559 |
|  | MSDP Y/N | 0.074 | 0.066 | 0.024 | 1.135 | 0.256 |
|  | heavy MSDP Y/N | 0.124 | 0.113 | 0.023 | 1.103 | 0.270 |

Criterion = Aggression age 18

| Model | R | R Square | Adjusted R Square | Std. Error | R Square Change | F Change | Sig. F Change |
| --- | --- | --- | --- | --- | --- | --- | --- |
| 1 | 0.079 | 0.006 | 0.006 | 0.996 | 0.006 | 14.868 | 0.000 |
| 2 | 0.081 | 0.007 | 0.006 | 0.996 | 0.000 | 0.450 | 0.502 |

*Note*: Model 1 = explored the role of MSDP, Model 2 = explored the effect of heavy MSDP above and beyond MSDP irrespective of quantity; 1 twin randomly selected out of every pair.

| Model | Predictors | B | Std. Error | Beta | t | p |
| --- | --- | --- | --- | --- | --- | --- |
| 1 | (Constant) | -0.026 | 0.022 |  | -1.161 | 0.246 |
|  | MSDP Y/N | 0.225 | 0.058 | 0.079 | 3.856 | 0 |
| 2 | (Constant) | -0.026 | 0.022 |  | -1.161 | 0.246 |
|  | MSDP Y/N | 0.201 | 0.069 | 0.071 | 2.93 | 0.003 |
|  | heavy MSDP Y/N | 0.078 | 0.117 | 0.016 | 0.671 | 0.502 |

**Table S11.** Meta-analysis exploring the effect of the association between MSDP and aggression after accounting for perinatal measures for males (a) and females (b) separately.

1. Females

|  | Correlation | Lower limit | Upper limit | Z-Value | p-Value |
| --- | --- | --- | --- | --- | --- |
| TEDS Age 9 | 0.118 | 0.068 | 0.167 | 4.648 | 0.000 |
| TEDS Age 12 | 0.054 | 0.000 | 0.108 | 1.950 | 0.051 |
| TEDS Age 16 | 0.084 | 0.024 | 0.142 | 2.767 | 0.006 |
| NTR Age 10 | 0.071 | 0.038 | 0.103 | 4.233 | 0.000 |
| NTR Age 12 | 0.045 | 0.009 | 0.081 | 2.437 | 0.015 |
| NTR Age 14 | 0.063 | 0.021 | 0.106 | 2.913 | 0.004 |
| NTR Age 16 | 0.045 | -0.011 | 0.100 | 1.564 | 0.118 |
| FT12 Age 12 | 0.089 | 0.045 | 0.134 | 3.907 | 0.000 |
| FT12 Age 14 | 0.032 | -0.068 | 0.130 | 0.624 | 0.533 |
| FT12 Age 17 | 0.045 | -0.021 | 0.110 | 1.340 | 0.180 |
| CATSS Age 9 | 0.071 | 0.045 | 0.096 | 5.445 | 0.000 |
| CATSS Age 15 | 0.032 | -0.013 | 0.076 | 1.390 | 0.165 |
| CATSS Age 18 | 0.089 | 0.039 | 0.139 | 3.494 | 0.000 |
| 3-level Random effects | 0.067 | 0.055 | 0.079 | 10.823 | 0.000 |

1. Males

|  | Correlation | Lower limit | Upper limit | Z-Value | p-Value |
| --- | --- | --- | --- | --- | --- |
| TEDS Age 9 | 0.071 | 0.019 | 0.123 | 2.660 | 0.008 |
| TEDS Age 12 | 0.045 | -0.014 | 0.104 | 1.495 | 0.135 |
| TEDS Age 16 | 0.055 | -0.014 | 0.123 | 1.573 | 0.116 |
| NTR Age 10 | 0.077 | 0.044 | 0.110 | 4.550 | 0.000 |
| NTR Age 12 | 0.077 | 0.041 | 0.113 | 4.153 | 0.000 |
| NTR Age 14 | 0.055 | 0.007 | 0.103 | 2.256 | 0.024 |
| NTR Age 16 | 0.045 | -0.023 | 0.112 | 1.304 | 0.192 |
| FT12 Age 12 | 0.071 | 0.014 | 0.128 | 2.423 | 0.015 |
| FT12 Age 14 | 0.032 | -0.061 | 0.124 | 0.677 | 0.499 |
| FT12 Age 17 | 0.105 | 0.038 | 0.171 | 3.082 | 0.002 |
| CATSS Age 9 | 0.084 | 0.059 | 0.109 | 6.607 | 0.000 |
| CATSS Age 15 | 0.032 | -0.016 | 0.080 | 1.294 | 0.196 |
| CATSS Age 18 | 0.145 | 0.087 | 0.202 | 4.852 | 0.000 |
| 3-level Random Effects | 0.073 | 0.059 | 0.087 | 10.189 | 0.000 |

**Table S12.** Meta-analysis exploring the effect of the association between perinatal measures (excluding MSDP) and aggression.

|  | Correlation | Lower limit | Upper limit | Z-Value | p-Value |
| --- | --- | --- | --- | --- | --- |
| TEDS Age 9 | 0.148 | 0.103 | 0.193 | 6.340 | 0.000 |
| TEDS Age 12 | 0.090 | 0.040 | 0.140 | 3.506 | 0.000 |
| TEDS Age 16 | 0.161 | 0.105 | 0.216 | 5.626 | 0.000 |
| NTR Age 10 | 0.167 | 0.144 | 0.190 | 14.160 | 0.000 |
| NTR Age 12 | 0.137 | 0.112 | 0.162 | 10.562 | 0.000 |
| NTR Age 14 | 0.126 | 0.095 | 0.157 | 7.810 | 0.000 |
| NTR Age 16 | 0.070 | 0.027 | 0.113 | 3.190 | 0.001 |
| FT12 Age 12 | 0.109 | 0.068 | 0.150 | 5.200 | 0.000 |
| FT12 Age 14 | 0.077 | 0.010 | 0.144 | 2.243 | 0.025 |
| FT12 Age 17 | 0.100 | 0.054 | 0.146 | 4.210 | 0.000 |
| CATSS Age 9 | 0.089 | 0.071 | 0.107 | 9.805 | 0.000 |
| CATSS Age 15 | 0.089 | 0.066 | 0.112 | 7.559 | 0.000 |
| CATSS Age 18 | 0.063 | 0.025 | 0.101 | 3.234 | 0.001 |
|  |  |  |  |  |  |
| Total Random Effects | 0.112 | 0.091 | 0.131 | 10.795 | 0.000 |

**Table S13.** Hierarchical regressions exploring the effects of paternal smoking in the NTR sample

1. Criterion: Aggression Age 10 (N = 6,897, one twin randomly selected out of each pair)

| Model | R | R Square | Adjusted R Square | Std. Error of the Estimate | R Square Change | F Change | df1 | df2 | Sig. F Change |
| --- | --- | --- | --- | --- | --- | --- | --- | --- | --- |
| 1. Early predictors | .167 | 0.028 | 0.027 | 0.974 | 0.028 | 39.642 | 5 | 6891 | 0.000 |
| 2. Paternal smoking DP | .179 | 0.032 | 0.031 | 0.972 | 0.004 | 30.103 | 1 | 6890 | 0.000 |
| **3. MSDP** | **.187** | **0.035** | **0.034** | **0.971** | **0.003** | **19.546** | **1** | **6889** | **0.000** |

| Model | Predictors | B | Std. Error | Beta | t | *p* |
| --- | --- | --- | --- | --- | --- | --- |
| 1 | (Constant) | 1.246 | 0.195 |  | 6.381 | 0.000 |
|  | maternal education | -0.094 | 0.016 | -0.080 | -5.838 | 0.000 |
|  | paternal education | -0.064 | 0.014 | -0.059 | -4.401 | 0.000 |
|  | maternal age | -0.021 | 0.003 | -0.083 | -6.801 | 0.000 |
|  | breastfeeding | -0.015 | 0.007 | -0.026 | -2.093 | 0.036 |
|  | gestation age | -0.003 | 0.005 | -0.006 | -0.532 | 0.595 |
| 2 | (Constant) | 1.043 | 0.198 |  | 5.260 | 0.000 |
|  | maternal education | -0.089 | 0.016 | -0.075 | -5.493 | 0.000 |
|  | paternal education | -0.049 | 0.015 | -0.045 | -3.345 | 0.001 |
|  | maternal age | -0.021 | 0.003 | -0.082 | -6.749 | 0.000 |
|  | breastfeeding | -0.013 | 0.007 | -0.023 | -1.811 | 0.070 |
|  | gestation age | -0.002 | 0.005 | -0.006 | -0.504 | 0.614 |
|  | **PSDP** | **0.085** | **0.016** | **0.067** | **5.487** | **0.000** |
| 3 | (Constant) | 0.922 | 0.200 |  | 4.610 | 0.000 |
|  | maternal education | -0.078 | 0.016 | -0.066 | -4.812 | 0.000 |
|  | paternal education | -0.047 | 0.015 | -0.043 | -3.178 | 0.001 |
|  | maternal age | -0.021 | 0.003 | -0.083 | -6.794 | 0.000 |
|  | breastfeeding | -0.010 | 0.007 | -0.018 | -1.441 | 0.150 |
|  | gestation age | -0.003 | 0.005 | -0.008 | -0.644 | 0.520 |
|  | PSDP | 0.061 | 0.017 | 0.048 | 3.672 | 0.000 |
|  | **MSDP** | **0.116** | **0.026** | **0.058** | **4.421** | **0.000** |

1. Criterion: Aggression Age 12 (N = 5,496, one twin randomly selected out of each pair)

| Model | R | R Square | Adjusted R Square | Std. Error of the Estimate | R Square Change | F Change | df1 | df2 | Sig. F Change |
| --- | --- | --- | --- | --- | --- | --- | --- | --- | --- |
| 1. Early predictors | .145 | 0.021 | 0.02 | 0.949926 | 0.021 | 23.551 | 5 | 5490 | 0.000 |
| 2. Paternal smoking DP | .151 | 0.023 | 0.022 | 0.949081 | 0.002 | 10.786 | 1 | 5489 | 0.001 |
| **3. MSDP** | **.162** | **0.026** | **0.025** | **0.947536** | **0.003** | **18.908** | **1** | **5488** | **0.000** |

| Model | Predictors | B | Std. Error | Beta | t | p |
| --- | --- | --- | --- | --- | --- | --- |
| 1 | (Constant) | 1.004 | 0.215 |  | 4.678 | 0.000 |
|  | maternal education | -0.056 | 0.018 | -0.049 | -3.183 | 0.001 |
|  | paternal education | -0.077 | 0.016 | -0.073 | -4.854 | 0.000 |
|  | maternal age | -0.021 | 0.003 | -0.083 | -6.036 | 0.000 |
|  | breastfeeding | 0.001 | 0.008 | 0.002 | 0.152 | 0.879 |
|  | gestation age | 0.000 | 0.005 | 0.000 | -0.022 | 0.982 |
| 2 | (Constant) | 0.861 | 0.219 |  | 3.939 | 0.000 |
|  | maternal education | -0.052 | 0.018 | -0.046 | -2.981 | 0.003 |
|  | paternal education | -0.067 | 0.016 | -0.064 | -4.153 | 0.000 |
|  | maternal age | -0.021 | 0.003 | -0.083 | -6.054 | 0.000 |
|  | breastfeeding | 0.002 | 0.008 | 0.004 | 0.307 | 0.759 |
|  | gestation age | 0.000 | 0.005 | 0.001 | 0.071 | 0.943 |
|  | **PSDP** | **0.057** | **0.017** | **0.045** | **3.284** | **0.001** |
| 3 | (Constant) | 0.732 | 0.220 |  | 3.320 | 0.001 |
|  | maternal education | -0.043 | 0.018 | -0.037 | -2.399 | 0.016 |
|  | paternal education | -0.064 | 0.016 | -0.061 | -3.970 | 0.000 |
|  | maternal age | -0.022 | 0.003 | -0.085 | -6.182 | 0.000 |
|  | breastfeeding | 0.005 | 0.008 | 0.010 | 0.682 | 0.496 |
|  | gestation age | 0.000 | 0.005 | 0.000 | -0.023 | 0.981 |
|  | PSDP | 0.031 | 0.018 | 0.025 | 1.709 | 0.087 |
|  | **MSDP** | **0.126** | **0.029** | **0.064** | **4.348** | **0.000** |

1. Criterion: Aggression Age 14 (N = 3,713, one twin randomly selected out of each pair)

| Model | R | R Square | Adjusted R Square | Std. Error of the Estimate | R Square Change | F Change | df1 | df2 | Sig. F Change |
| --- | --- | --- | --- | --- | --- | --- | --- | --- | --- |
| 1. Early predictors | .128 | 0.016 | 0.015 | 0.980 | 0.016 | 12.416 | 5 | 3707 | 0.000 |
| 2. Paternal smoking DP | .141 | 0.02 | 0.018 | 0.978 | 0.003 | 13.094 | 1 | 3706 | 0.000 |
| **3. MSDP** | **.147** | **0.022** | **0.02** | **0.978** | **0.002** | **6.13** | **1** | **3705** | **0.013** |

| Model | Predictors | B | Std. Error | Beta | t | p |
| --- | --- | --- | --- | --- | --- | --- |
| 1 | (Constant) | 0.078 | 0.272 |  | 0.285 | 0.775 |
|  | maternal education | -0.060 | 0.022 | -0.050 | -2.672 | 0.008 |
|  | paternal education | -0.089 | 0.020 | -0.081 | -4.437 | 0.000 |
|  | maternal age | -0.010 | 0.004 | -0.040 | -2.388 | 0.017 |
|  | breastfeeding | 0.026 | 0.010 | 0.046 | 2.699 | 0.007 |
|  | gestation age | 0.017 | 0.007 | 0.041 | 2.486 | 0.013 |
| 2 | (Constant) | -0.113 | 0.277 |  | -0.407 | 0.684 |
|  | maternal education | -0.055 | 0.022 | -0.046 | -2.471 | 0.014 |
|  | paternal education | -0.075 | 0.020 | -0.069 | -3.683 | 0.000 |
|  | maternal age | -0.010 | 0.004 | -0.040 | -2.392 | 0.017 |
|  | breastfeeding | 0.028 | 0.010 | 0.048 | 2.855 | 0.004 |
|  | gestation age | 0.017 | 0.007 | 0.042 | 2.560 | 0.011 |
|  | **PSDP** | **0.078** | **0.022** | **0.061** | **3.619** | **0.000** |
| 3 | (Constant) | -0.203 | 0.279 |  | -0.727 | 0.467 |
|  | maternal education | -0.048 | 0.023 | -0.040 | -2.121 | 0.034 |
|  | paternal education | -0.073 | 0.021 | -0.066 | -3.566 | 0.000 |
|  | maternal age | -0.011 | 0.004 | -0.041 | -2.432 | 0.015 |
|  | breastfeeding | 0.029 | 0.010 | 0.052 | 3.038 | 0.002 |
|  | gestation age | 0.016 | 0.007 | 0.040 | 2.457 | 0.014 |
|  | PSDP | 0.060 | 0.023 | 0.047 | 2.653 | 0.008 |
|  | **MSDP** | **0.094** | **0.038** | **0.044** | **2.476** | **0.013** |

1. Criterion: Aggression Age 16 (N = 2024, one twin randomly selected out of each pair)

| Model | R | R Square | Adjusted R Square | Std. Error of the Estimate | R Square Change | F Change | df1 | df2 | Sig. F Change |
| --- | --- | --- | --- | --- | --- | --- | --- | --- | --- |
| 1. Early predictors | .061a | 0.004 | 0.001 | 0.29166 | 0.004 | 1.523 | 5 | 2018 | 0.179 |
| 2. Paternal smoking DP | .084b | 0.007 | 0.004 | 0.29125 | 0.003 | 6.688 | 1 | 2017 | 0.01 |
| 3. MSDP | .088c | 0.008 | 0.004 | 0.29123 | 0.001 | 1.25 | 1 | 2016 | 0.264 |

| Model | Predictors | B | Std. Error | Beta | t | p |
| --- | --- | --- | --- | --- | --- | --- |
| 1 | (Constant) | 0.202 | 0.108 |  | 1.866 | 0.062 |
|  | maternal education | -0.018 | 0.009 | -0.050 | -1.951 | 0.051 |
|  | paternal education | -0.005 | 0.008 | -0.014 | -0.576 | 0.565 |
|  | maternal age | 0.000 | 0.002 | -0.001 | -0.040 | 0.968 |
|  | breastfeeding | 0.002 | 0.004 | 0.013 | 0.567 | 0.571 |
|  | gestation age | 0.003 | 0.003 | 0.026 | 1.157 | 0.247 |
| 2 | (Constant) | 0.143 | 0.111 |  | 1.290 | 0.197 |
|  | maternal education | -0.017 | 0.009 | -0.047 | -1.848 | 0.065 |
|  | paternal education | 0.000 | 0.008 | -0.001 | -0.025 | 0.980 |
|  | maternal age | 0.000 | 0.002 | 0.000 | 0.017 | 0.987 |
|  | breastfeeding | 0.003 | 0.004 | 0.017 | 0.732 | 0.464 |
|  | gestation age | 0.003 | 0.003 | 0.027 | 1.216 | 0.224 |
|  | **PSDP** | **0.023** | **0.009** | **0.060** | **2.586** | **0.010** |
| 3 | (Constant) | 0.126 | 0.112 |  | 1.131 | 0.258 |
|  | maternal education | -0.015 | 0.009 | -0.043 | -1.692 | 0.091 |
|  | paternal education | 0.000 | 0.008 | 0.000 | -0.006 | 0.995 |
|  | maternal age | 0.000 | 0.002 | 0.000 | -0.012 | 0.990 |
|  | breastfeeding | 0.003 | 0.004 | 0.020 | 0.855 | 0.393 |
|  | gestation age | 0.003 | 0.003 | 0.027 | 1.187 | 0.235 |
|  | PSDP | 0.019 | 0.009 | 0.051 | 2.072 | 0.038 |
|  | MSDP | 0.017 | 0.015 | 0.027 | 1.118 | 0.264 |

**Table S14.** Hierarchical regressions exploring the effects of paternal smoking in the FT12 sample

1. Criterion: Aggression Age 12 (N = 1,330, 1 twin randomly selected out of each pair)

| Model | R | R Square | Adjusted R Square | Std. Error of the Estimate | R Square Change | F Change | df1 | df2 | Sig. F Change |
| --- | --- | --- | --- | --- | --- | --- | --- | --- | --- |
| 1. Early predictors | .090a | 0.008 | 0.005 | 0.319 | 0.008 | 2.730 | 4 | 1325 | 0.028 |
| 2. Father ever smoked | .108b | 0.012 | 0.008 | 0.319 | 0.003 | 4.591 | 1 | 1324 | 0.032 |
| **3 MSDP** | **.128c** | **0.016** | **0.012** | **0.318** | **0.005** | **6.472** | **1** | **1323** | **0.011** |

| Model | Predictors | B | Std. Error | Beta | t | p |
| --- | --- | --- | --- | --- | --- | --- |
| 1 | (Constant) | 0.892 | 0.161 |  | 5.553 | 0 |
|  | maternal education | -0.006 | 0.002 | -0.087 | -3.186 | 0.001 |
|  | paternal education | -0.001 | 0.003 | -0.007 | -0.231 | 0.817 |
|  | maternal age | -0.001 | 0.003 | -0.01 | -0.329 | 0.742 |
|  | gestation age | 0.003 | 0.004 | 0.019 | 0.711 | 0.477 |
| 2 | (Constant) | 0.805 | 0.165 |  | 4.867 | 0 |
|  | maternal education | -0.006 | 0.002 | -0.085 | -3.122 | 0.002 |
|  | paternal education | 8.16E-05 | 0.003 | 0.001 | 0.031 | 0.975 |
|  | maternal age | -0.001 | 0.003 | -0.008 | -0.262 | 0.794 |
|  | gestation age | 0.003 | 0.004 | 0.022 | 0.803 | 0.422 |
|  | paternal smoking | 0.064 | 0.03 | 0.059 | 2.143 | 0.032 |
| 3 | (Constant) | 0.634 | 0.178 |  | 3.556 | 0 |
|  | maternal education | -0.006 | 0.002 | -0.084 | -3.063 | 0.002 |
|  | paternal education | 0 | 0.003 | 0.003 | 0.1 | 0.921 |
|  | maternal age | 0 | 0.003 | 0.004 | 0.138 | 0.89 |
|  | gestation age | 0.004 | 0.004 | 0.026 | 0.942 | 0.346 |
|  | Paternal smoking | 0.061 | 0.03 | 0.056 | 2.029 | 0.043 |
|  | **MSDP** | **0.125** | **0.049** | **0.071** | **2.544** | **0.011** |

1. Criterion Aggression age 17 (N = 1116; 1 twin randomly selected out of each pair. Note that the analyses on aggression at age 14 were not included as the association was not significant in the first place)

| Model | R | R Square | Adjusted R Square | Std. Error of the Estimate | R Square Change | F Change | df1 | df2 | Sig. F Change |
| --- | --- | --- | --- | --- | --- | --- | --- | --- | --- |
| 1. Early predictors | .105a | 0.011 | 0.008 | 1.008688 | 0.011 | 3.262 | 4 | 1161 | 0.011 |
| 2. Father ever smoked | .106b | 0.011 | 0.007 | 1.009114 | 0.000 | 0.021 | 1 | 1160 | 0.886 |
| 3 MSDP | .119c | 0.014 | 0.009 | 1.007954 | 0.003 | 3.672 | 1 | 1159 | 0.056 |

| Model | Predictors | B | Std. Error | Beta | t | p |
| --- | --- | --- | --- | --- | --- | --- |
| 1 | (Constant) | 0.689 | 0.552 |  | 1.249 | 0.212 |
|  | maternal education | -0.015 | 0.006 | -0.072 | -2.47 | 0.014 |
|  | paternal education | -0.003 | 0.009 | -0.011 | -0.347 | 0.729 |
|  | maternal age | -0.02 | 0.009 | -0.069 | -2.115 | 0.035 |
|  | gestation age | 0.009 | 0.013 | 0.02 | 0.673 | 0.501 |
| 2 | (Constant) | 0.71 | 0.571 |  | 1.244 | 0.214 |
|  | maternal education | -0.015 | 0.006 | -0.072 | -2.472 | 0.014 |
|  | paternal education | -0.003 | 0.009 | -0.012 | -0.362 | 0.717 |
|  | maternal age | -0.02 | 0.009 | -0.069 | -2.115 | 0.035 |
|  | gestation age | 0.008 | 0.013 | 0.019 | 0.662 | 0.508 |
|  | Paternal smoking | -0.015 | 0.104 | -0.004 | -0.144 | 0.886 |
| 3 | (Constant) | 0.279 | 0.613 |  | 0.455 | 0.649 |
|  | maternal education | -0.015 | 0.006 | -0.072 | -2.473 | 0.014 |
|  | paternal education | -0.003 | 0.009 | -0.01 | -0.287 | 0.774 |
|  | maternal age | -0.017 | 0.009 | -0.06 | -1.812 | 0.07 |
|  | gestation age | 0.01 | 0.013 | 0.023 | 0.771 | 0.441 |
|  | paternal smoking | -0.024 | 0.104 | -0.007 | -0.233 | 0.816 |
|  | MSDP | 0.324 | 0.169 | 0.057 | 1.916 | 0.056 |

**Table S15** Association between MSDP and aggression after accounting for aggressive parenting at ages 9(a) and 12 (b) in the TEDS sample.

1. Criterion aggression at age 9 (N = 1,740, 1 twin out of each pair randomly selected)

| Model | R | R Square | Adjusted R Square | Std. Error of the Estimate | R Square Change | F Change | df1 | df2 | Sig. F Change |
| --- | --- | --- | --- | --- | --- | --- | --- | --- | --- |
| 1. Early predictors | .170 | 0.029 | 0.024 | 0.9638 | 0.029 | 5.784 | 9 | 1740 | 0.000 |
| 2. Aggressive parenting | .240 | 0.058 | 0.052 | 0.9498 | 0.029 | 53.005 | 1 | 1739 | 0.000 |
| **3. MSDP** | **.251** | **0.063** | **0.057** | **0.9473** | **0.005** | **10.081** | **1** | **1738** | **0.002** |

| Predictors | B | Std. Error | Beta | t | p |
| --- | --- | --- | --- | --- | --- |
| (Constant) | -0.329 | 0.494 |  | -0.666 | 0.506 |
| maternal age | 0.005 | 0.008 | 0.023 | 0.654 | 0.513 |
| paternal age | -0.004 | 0.005 | -0.024 | -0.803 | 0.422 |
| paternal education | -0.005 | 0.018 | -0.011 | -0.258 | 0.797 |
| maternal education | 0.014 | 0.019 | 0.029 | 0.709 | 0.479 |
| drank while pregnant | 0.020 | 0.034 | 0.014 | 0.577 | 0.564 |
| length of breastfeeding | 0.000 | 0.000 | -0.037 | -1.526 | 0.127 |
| birthweight | 0.000 | 0.000 | -0.034 | -1.061 | 0.289 |
| gestational age | 0.009 | 0.013 | 0.022 | 0.690 | 0.490 |
| stress during pregnancy | 0.133 | 0.057 | 0.055 | 2.324 | 0.020 |
| low SES | 0.130 | 0.066 | 0.128 | 1.959 | 0.050 |
| aggressive/harsh parenting | 0.426 | 0.059 | 0.170 | 7.179 | 0.000 |
| **smoking while pregnant** | **0.192** | **0.062** | **0.075** | **3.083** | **0.002** |

1. Criterion: Aggression age 12 (N = 2,886, 1 twin out of each pair randomly selected)

| Model | R | R Square | Adjusted R Square | Std. Error | R Square Change | F Change | df1 | df2 | Sig. F Change |
| --- | --- | --- | --- | --- | --- | --- | --- | --- | --- |
| 1. Early predictors | .120a | 0.014 | 0.010 | 0.971 | 0.014 | 2.982 | 10 | 2028 | 0.001 |
| 2. Aggressive parenting | .147b | 0.022 | 0.016 | 0.967 | 0.007 | 14.887 | 1 | 2027 | 0.000 |
| **3. MSDP** | **.155c** | **0.024** | **0.018** | **0.966** | **0.003** | **5.201** | **1** | **2026** | **0.023** |

| Predictors | B | Std. Error | Beta | t | p |
| --- | --- | --- | --- | --- | --- |
| (Constant) | -0.677 | 0.458 |  | -1.478 | 0.140 |
| maternal age | 0.004 | 0.008 | 0.017 | 0.502 | 0.616 |
| paternal age | -0.001 | 0.005 | -0.004 | -0.161 | 0.872 |
| paternal education | 0.000 | 0.017 | -0.001 | -0.027 | 0.979 |
| maternal education | 0.013 | 0.018 | 0.027 | 0.725 | 0.469 |
| drank while pregnant | 0.014 | 0.031 | 0.010 | 0.448 | 0.654 |
| length of breastfeeding | 0.000 | 0.000 | -0.056 | -2.442 | 0.015 |
| birth weight | 0.000 | 0.000 | 0.044 | 1.449 | 0.148 |
| gestational age | 0.008 | 0.013 | 0.020 | 0.656 | 0.512 |
| stress during pregnancy | 0.034 | 0.054 | 0.014 | 0.628 | 0.530 |
| low SES | 0.094 | 0.063 | 0.090 | 1.496 | 0.135 |
| aggressive/harsh parenting | 0.291 | 0.074 | 0.087 | 3.939 | 0.000 |
| **smoking while pregnant** | **0.149** | **0.065** | **0.052** | **2.280** | **0.023** |

**Measure of MSDP across samples.**

In **TEDS**, mothers reported how much they smoked during their twin pregnancy on the following three levels: 0 = none; 1 = 10 cigarettes per day or less; and 2 = 11 cigarettes per day or more. In **NTR**, the measure of MSDP originally included four responses (1= did not smoke during pregnancy, 2 = smoked a pipe, 3 = smoked less than 10 cigarettes per day, and 4 = smoked 10 or more cigarettes per day). As response item 2 (smoked a pipe) only included few participants, the MSDP measure was recoded to include 3 levels (1 = did not smoke; 2 = smoked pipe or less than 10 cigarettes per day; and 3 = smoked 10 or more cigarettes per day). In **FT12**, the MSDP measure asked mothers how many cigarettes per day, if any, they had smoked while pregnant. Mothers reported the average number of cigarettes they smoked every day while pregnant. This measure was recoded into (1 = none; 2 = less than 10 cigarettes per day and 3 = more than 10 cigarettes per day). In **CATSS**, MSDP assessment was based on self-report of daily tobacco use at the first antenatal visit, which typically occurred during the first trimester. Information on MSDP during the second trimester was also available in a subsample (*N* = 6,484). Overall, mothers answered two questions about smoking during pregnancy: (1) did you smoke during the first trimester of your pregnancy? And (2) did you smoke during the second trimester of your pregnancy? For each question mothers could answer NO, coded as 1; YES, less than 10 cigarettes per day, coded as 2; and YES, more than 10 cigarettes per day, coded as 3. A composite of questions 2 and 3, generally describing smoking during pregnancy, was created in order to measure MSDP. In the CATSS sample, information on smoking heavily was obtained combining reports across the first and second trimester.

**Example of the R code used to run the three-level meta-analyses**

library (metafor)

library (dplyr)

dat <- read.csv()

###transform the r value ri into a z score (vi) and add a vi (variance) column to the data frame

dat = escalc(measure="ZCOR", ri=ri,ni=ni, data=dat)

###Level 2: Random Effects meta-analysis ###

Res <- rma(yi, vi, data=dat)

res

predict (res, digits=3, transf=transf.ztor)

confint (res)

###Level 3: 3-level meta-analysis accounting for the clustering into twin cohorts ###

multi.meta <- rma.mv(yi, vi, data= dat, random= list(~1|esid, ~1|studyid), method="REML")

multi.meta

summary(multi.meta)

#obtain CIs for tau^2, tau, I^2, H^2

confint(multi.meta)

### Calculating the proportion of true heterogeneity due to between and within sample heterogeneity:

W <- diag(1/dat$vi)

X <- model.matrix(multi.meta)

P <- W - W %*% X %*% solve(t(X) %*% W %*% X) %*% t(X) %*% W

100 * sum(multi.meta$sigma2) / (sum(multi.meta$sigma2) + (multi.meta$k-multi.meta$p)/sum(diag(P))) #total

100 * multi.meta$sigma2 / (sum(multi.meta$sigma2) + (multi.meta$k-multi.meta$p)/sum(diag(P))) #separate
